# Disentangling temporal associations in marine microbial networks

**DOI:** 10.1186/s40168-023-01523-z

**Published:** 2023-04-21

**Authors:** Ina Maria Deutschmann, Anders K. Krabberød, Francisco Latorre, Erwan Delage, Cèlia Marrasé, Vanessa Balagué, Josep M. Gasol, Ramon Massana, Damien Eveillard, Samuel Chaffron, Ramiro Logares

**Affiliations:** 1 Institute of Marine Sciences (ICM), CSIC, Passeig Marítim de La Barceloneta, 37-49, 08003 Barcelona, Spain; 2 Department of Biosciences/Section for Genetics and Evolutionary Biology (EVOGENE), University of Oslo, p.b. 1066 Blindern, N-0316 Oslo, Norway; 3 Nantes Université, École Centrale Nantes, CNRS, LS2N, UMR 6004, F-44000 Nantes, France; 4 Research Federation for the Study of Global Ocean Systems Ecology and Evolution, FR2022/Tara Oceans GOSEE, F-75016 Paris, France

**Keywords:** Association network, Temporal network, Time series, Microbial interactions, Microorganisms, Ocean, Plankton

## Abstract

**Background:**

Microbial interactions are fundamental for Earth’s ecosystem functioning and biogeochemical cycling. Nevertheless, they are challenging to identify and remain barely known. Omics-based censuses are helpful in predicting microbial interactions through the statistical inference of single (static) association networks. Yet, microbial interactions are dynamic and we have limited knowledge of how they change over time. Here, we investigate the dynamics of microbial associations in a 10-year marine time series in the Mediterranean Sea using an approach inferring a time-resolved (temporal) network from a single static network.

**Results:**

A single static network including microbial eukaryotes and bacteria was built using metabarcoding data derived from 120 monthly samples. For the decade, we aimed to identify persistent, seasonal, and temporary microbial associations by determining a temporal network that captures the interactome of each individual sample. We found that the temporal network appears to follow an annual cycle, collapsing, and reassembling when transiting between colder and warmer waters. We observed higher association repeatability in colder than in warmer months. Only 16 associations could be validated using observations reported in literature, underlining our knowledge gap in marine microbial ecological interactions.

**Conclusions:**

Our results indicate that marine microbial associations follow recurrent temporal dynamics in temperate zones, which need to be accounted for to better understand the functioning of the ocean microbiome. The constructed marine temporal network may serve as a resource for testing season-specific microbial interaction hypotheses. The applied approach can be transferred to microbiome studies in other ecosystems.

Video Abstract

**Supplementary Information:**

The online version contains supplementary material available at 10.1186/s40168-023-01523-z.

## Introduction

Microorganisms are the most abundant life forms on Earth, being fundamental for global ecosystem functioning [1–3]. The total number of microorganisms on the planet is estimated to be ≈ 10^12^ species [4] and ≈ 10^30^ cells [5, 6]. In particular, microorganisms dominate the largest biome, the ocean, which harbors ≈ 10^29^ microbial cells [6] accounting for ~ 70% of the total marine biomass [7, 8].

Microbial communities are highly dynamic, and their composition is determined through a combination of ecological processes: selection, dispersal, drift, and speciation [9]. Selection is a prominent community structuring force exerted via multiple abiotic and biotic factors [10, 11]. Several studies have addressed the role of *abiotic* factors in structuring microbial communities. For example, the temperature is one of the main factors exerting selection in the ocean microbiome over spatiotemporal scales [12–15]. *Biotic* factors can also strongly affect microbial communities [16]. However, a mechanistic understanding of how they affect community structure is currently lacking, as the diversity of microbial interactions is barely known [3, 17].

The vast microbial diversity and the fact that most microorganisms are still uncultured [18, 19] make it impossible to experimentally test all potential interactions between pairs of microbes. However, omics-technologies allow estimating microbial relative abundances over spatiotemporal scales, which permits determining statistical associations between taxa. These associations can be summarized as a network with nodes representing microorganisms and edges representing potential interactions [20, 21].

As microorganisms are highly interconnected [21], association networks provide a general overview of the entire microbial system and have been tremendously valuable for generating novel hypotheses about putative interactions. In particular, time series have allowed identifying potential ecological interactions among marine microorganisms [22–28]. For example, previous work characterized ecological links between marine archaea, bacteria, and eukaryotes [22], including links with viruses [24, 26], also investigating within- and between ocean-depth relationships [25, 27]. These studies not only identified time-dependent associations among ecologically important taxa, but also potential synergistic or antagonistic relationships, as well as possible “keystone” species and potential niches [22, 23]. Moreover, several studies have reported more associations among microorganisms than between microorganisms and environmental variables, suggesting the importance of biotic relationships in structuring microbial community assemblages [22, 28].

Previous studies have used temporal microbial abundance data to infer static networks summarizing all potential associations in space and time. This static abstraction assumes that the network topology does not change (static) and edges represent persistent associations assumed as interactions [29]; that is, edges are present throughout time and space. This assumption cannot represent the reality of most microbial interactions. Thus, a single static network usually contains persistent, temporary, and recurring (including seasonal) associations that need to be disentangled.

Despite the contribution of static networks to our understanding of microbial interactions in the ocean, it is necessary to incorporate the temporal dimension. Using a time-resolved, i.e., temporal network instead of a single static network would allow investigation of the dynamic nature of microbial associations and how they change over time, whether the change is deterministic or stochastic, and how environmental selection influences network architecture. Addressing these questions is fundamental for a better understanding of the dynamic interactions that underpin ecosystem function in the ocean. Here, we investigated marine microbial associations through time by determining a temporal network from a single static network.

## Materials and methods

### The Blanes Bay Microbial Observatory (BBMO)

The BBMO is a coastal oligotrophic site in the North-Western Mediterranean Sea (41^*◦*^ 40′ N, 2^*◦*^ 48′ E) without major natural disturbances and little anthropogenic pressure, except for the construction of a nearby harbor between 2010 and 2012 [30, 31]. The seasonal cycle is typical for a temperate coastal system [30], and the main environmental factors influencing seasonal microbial succession have been well studied and are known [12]. Shortly, the water column is slightly stratified in summer before it destabilizes and mixes with water from offshore in late fall, increasing the availability of inorganic nutrients with maximum concentrations in winter, between November and March. The high amount of nutrients and increasing light induce phytoplankton blooms, mostly in late winter-early spring. During summer, inorganic nutrients become limiting, the primary production is minimal, and dissolved organic carbon accumulates [30].

### From sampling to microbial relative abundances

We sampled surface water (*≈* 1 m depth) monthly from January 2004 to December 2013 to determine microbial community composition and also measured ten environmental variables, which were previously described [13, 30]: water temperature (^◦^C) and salinity (obtained in situ with a SAIV-AS-SD204 Conductivity-Temperature-Depth probe), day-length (hours of light), turbidity (Secchi depth in meters), total chlorophyll-a concentration (*µ*g/l, fluorometry of acetone extracts after 150 ml filtration on GF/F filters [30]), and five inorganic nutrients: PO_4_^3*−*^, NH_4_^+^, NO_2_^*−*^, NO_3_^*−*^, and SiO_2_ (*µ*M, determined with an Alliance Evolution II autoanalyzer [32]).

Sampling of microbial communities, DNA extraction, rRNA-gene amplification, sequencing, and bioinformatic analyses are explained in detail in [28]. In short, 6 L of water were prefiltered through a 200-µm nylon mesh and subsequently filtered through another 20-µm nylon mesh and separated into nanoplankton (3*–*20 µm) and picoplankton (0*.*2*–*3 µm) using a 3-µm and 0.2-µm pore-size polycarbonate and Sterivex filters, respectively. Then, the DNA was extracted from the filters using a phenol–chloroform protocol [33], which has been modified for purification with Amicon units (Millipore). We amplified the 18S rRNA genes (V4 region) with the primers TAReukFWD1 and TAReukREV3 [34], and the 16S rRNA genes (V4 region) with Bakt 341F [35] and 806RB [36]. Amplicons were sequenced in a MiSeq platform (2 × 250 bp) at RTL Genomics (Lubbock, Texas). Read quality control, trimming, and inference of operational taxonomic units (OTUs) delineated as amplicon sequence variants (ASVs) were done with DADA2 [37], v1.10.1, with the maximum number of expected errors set to 2 and 4 for the forward and reverse reads, respectively.

Microbial sequence abundance tables were obtained for each size fraction for both microbial eukaryotes and prokaryotes. Before merging the tables, we subsampled each table to the lowest sequencing depth of 4907 reads with the *rrarefy* function from the Vegan R-package [38], v2.4–2, (see details in [28]). We excluded 29 nanoplankton samples (March 2004, February 2005, May 2010–July 2012) due to suboptimal amplicon sequencing. In these samples, abundances were estimated using seasonally aware missing value imputation by the weighted moving average for time series as implemented in the *imputeTS* R-package, v2.8 [39]. These imputed values did not introduce biases in the analyses [28].

Sequence taxonomy was inferred using the naïve Bayesian classifier method [40] together with the SILVA database [41], v.132, as implemented in DADA2 [37]. Additionally, eukaryotic microorganisms were BLASTed [42] against the Protist Ribosomal Reference (PR2) database [43], v4.10.0. The PR2 classification was used when the taxonomic assignment from SILVA and PR2 disagreed. We removed ASVs that were identified as Metazoa, Streptophyta, plastids, mitochondria, and Archaea since the 341F-primer is not optimal for recovering this domain [44]. Besides, Haptophyta is known to be missed by the primer TAReukREV3 [45].

The resulting table contained 2924 ASVs (Table 1A). Next, we removed rare ASVs keeping ASVs with sequence abundance sums above 100 reads and prevalence above 15% of the samples, i.e., we considered taxa present in at least 19 months. The resulting table contained 1782 ASVs (Table 1B). An ASV can appear twice, in the nanoplankton and picoplankton size fractions. However, an ASV may be detected in both size fractions due to dislodging cells or particles and filter clogging, which can introduce biases in our analysis. To reduce these biases, and as done previously [28], we divided the abundance sum of the larger by the smaller size fraction for each ASV appearing in both size fractions and set the picoplankton abundances to zero if the ratio exceeded 2. Likewise, we set the nanoplankton abundances to zero if the ratio was below 0*.*5. This operation removed two eukaryotic ASVs and 41 bacterial ASVs from the nanoplankton, and 30 bacterial ASVs from the picoplankton (Table 1C). The resulting abundance table was used for network inference.

**Table 1:** Number and fraction of ASVs and reads (total, bacterial, and eukaryotic) for the sequence abundance tables (A, B, and C), the preliminary network with significant edges (D), and the single static network (E) obtained after removing environmentally driven edges and edges with association partners appearing more often alone than with the partner. If an ASV appeared in the nano- and pico-plankton size fractions, it was counted twice

**Count tables**	**ASVs**	**Reads**	**Eukaryote**	**Eukaryotic reads**	**Bacteria**	**Bacterial reads**
A	2924	2,273,548	1365	1,121,855	1559	1,151,693
B	1782	2,155,318	1009	1,057,599	773	1,097,719
C	1709	2,062,866	1007	1,057,263	702	1,005,603
D	754	1,657,885	306	730,025	448	927,860
E	709	1,621,959	294	719,558	415	902,401
**Fractions**	**ASV**	**Reads**	**Eukaryote**	**Eukaryotic reads**	**Bacteria**	**Bacterial reads**
B/A*100	60.94	94.80	73.92	94.27	49.58	95.31
C/A*100	58.45	90.73	73.77	94.24	45.03	87.32
D/C*100	44.12	80.37	30.39	69.05	69.05	92.27
E/C*100	41.49	78.63	29.20	68.06	59.12	89.74

*A* raw sequence abundance table,* B* sequence abundance table without rare ASVs,* C* sequence abundance table after size-fraction filtering,* D* preliminary network with significant edges,* E* single static network

### From sequence abundances to the single static network

First, we constructed a preliminary network using the tool eLSA [46, 47], as done in [28, 48], including default normalization and *z*-score transformation, using median and median absolute deviation. Although we are aware of time-delayed interactions, we considered our 1-month sampling interval too large for inferring time-delayed associations with a solid ecological basis and focused on contemporary interactions between co-occurring microorganisms. Using 2000 iterations, we estimated *p* values with a mixed approach that performs a random permutation test of a co-occurrence if the comparison’s theoretical *p* values are below 0*.*05. The Bonferroni false discovery rate (*q*) was calculated based on the *p* values using the *p.adjust* function from the stats R-package [49]. We used the 0.001 significance threshold for the *p* and *q* values, as suggested in other studies [20]. We refrained from using an association strength threshold since it may not be appropriate to differentiate between true interactions and environmentally-driven associations [48]. Furthermore, changing thresholds have been shown to lead to different network properties [50]. The preliminary network contained 754 nodes and 29,820 edges (24,458, 82% positive, and 5362, 18% negative).

Second, for environmentally driven edge detection, we applied EnDED [48], combining the methods interaction information (with a 0.05 significance threshold and 10,000 iterations) and data processing inequality. We inserted artificial edges connecting each node to each environmental parameter. We identified and removed 3315 (11.12%) edges that were environmentally driven; 26,505 edges (23,405, 88.3% positive, and 3100, 11.7% negative) remained (Supplementary Tables 3 and 4).

Third, we determined the Jaccard index, $J$, for each microorganisms pair associated through an edge, in order to remove associations between microorganisms that have a low co-occurrence. Let ${S}_{i}$ be the set of samples in which both microorganisms are present (sequence abundance above zero), and ${S}_{u}$ be the set of samples in which one or both microorganisms are present. Then, we can calculate the Jaccard index as the fraction of samples in which both appear (intersection) from the number of samples in which at least one appears (union): ${J=S}_{i}/{S}_{u}$. We chose $J>0.5$ as in previous work [48], which removed 9879 edges and kept 16,626 edges (16,481, 99.1% positive and 145, 0.9% negative). We removed isolated nodes, i.e., nodes without an associated partner in the network. The number and fraction of retained reads are listed in Table 1. The resulting network is our single static network.

### From the single static network to the temporal network

**Fig. 1:**
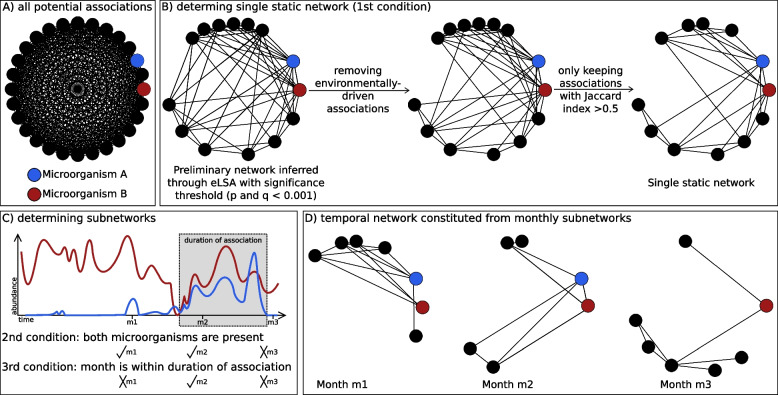
Estimating a temporal network from a single static network via subnetworks. **A** A complete network would contain all possible associations (edges) between microorganism (nodes). **B** The single static network inferred with the network construction tool eLSA and the applied filtering strategy considering association significance, the removal of environmentally driven associations, and associations whose partners appeared in more samples together than alone, i.e., Jaccard index being above 0.5. An association having to be present in the single static network is the first out of the three conditions for an association to be present in a monthly subnetwork. **C** In order to determine monthly subnetworks, we established two further conditions for each edge. First, both microorganisms need to be present in the sample taken in the specific month. Second, the month lays within the time window of the association inferred through the network construction tool. Here, 3 months are indicated as an example. **D** Example of monthly subnetworks for the 3 months. The colored nodes correspond to the abundances depicted in **C**

We determined the temporal network comprising 120 sample-specific (monthly) subnetworks through the three conditions indicated below and visualized in Fig. 1. The subnetworks are derived from the single static network and contain a node subset and an edge subset of the static network. Let *e* be an association between microorganisms *A* and *B*, with association duration *d* = (*t*_1_*, t*_2_), i.e., the association starts at time point *t*_1_ and ends at *t*_2_. Then, considering month *m*, the association *e* is present in the monthly subnetwork *N*_*m*_, if:

1. *e* is an association in the single static network
2. The microorganisms *A* and *B* are present within month *m*
3. *m* is within the duration of association, i.e., *t*_1_ ≤ *m* ≤ *t*_2_.

With the second condition, we assumed that an association was present in a month if both microorganisms were present, i.e., the microbial abundances were non-zero for that month. However, we cannot assume that microbial co-occurrence is a sufficient condition for a microbial interaction because different mechanisms influence species and interactions, and the environmental filtering of species and interactions can differ [51]. Using only the species occurrence assumption would increase association prevalence. To lower this bias, we also required that the association was present in the static network, first condition, and within the association duration, third condition, both inferred by eLSA [46, 47]. Lastly, we removed isolated nodes from each monthly subnetwork.

### Network analysis

We computed global network metrics to characterize the single static network and each monthly subnetwork using the igraph R-package [52]. Some metrics tend to be more correlated than others implying redundancy between them, grouping them into four groups [53]. Thus, we selected one metric from each group: *edge density*, *average path length*, *transitivity*, and *assortativity* based on node degree. In addition, we also computed the *average strength of positive associations* between microorganisms using the mean, and *assortativity* based on the nominal classification of nodes into bacteria and eukaryotes. Assortativity (bacteria vs. eukaryotes) is positive if bacteria tend to connect with bacteria and eukaryotes tend to connect with eukaryotes. It is negative if bacteria tend to connect to eukaryotes and vice versa. We also quantified associations by calculating their prevalence as the fraction of monthly subnetworks in which the association was present for all 10 years (recurrence) and monthly. We visualized highly prevalent associations with the *circlize* R-package [54]. We tested our hypotheses of environmental factors influencing network topology by calculating the Spearman correlations between global network metrics and environmental data. We used Holm’s multiple test correction to adjust *p* values [55], with the function *corr.test* in the *psych* R-package [56]. We used Gephi [57], v.0.9.2, and the Fruchterman Reingold Layout [58] for network visualizations.

### Test of network construction tool

We have used eLSA to estimate the duration of an association, which we used as the third condition (*m* is within the duration of association, i.e., *t*_1_ ≤ *m* ≤ *t*_2_) to infer the sample-specific subnetworks. Other methods may perform better on compositional data such as ours [59] (although this is not necessarily the case; see [60]). Therefore, we tested another network construction approach (FlashWeave [61]) for comparative purposes. FlashWeave performed better than eLSA in some benchmark tests run by other authors, while eLSA performed better than FlashWeave in other tests [61]. FlashWeave can handle sparse datasets taking zeros into account and avoiding spurious correlations between ASVs that share many zeros. However, it neglects the temporal variation. To control data compositionality [59], we applied a centered-log-ratio transformation separately to the bacterial and eukaryotic read abundance tables before merging them. Then, we inferred a network using FlashWeave [61], selecting the options “heterogeneous” and “sensitive.” We have run analyses including the environmental data (10 variables; see above). The resulting network had 932 nodes and 1440 edges. Next, we determined a temporal network using conditions (1) and (2) but not (3) since the temporal duration is not estimated by FlashWeave. FlashWeave results are used hereafter to compare against eLSA, although eLSA is kept as the main network construction tool in our work, given that it allows determination of the duration of the associations and there is no evidence suggesting a poor performance of this tool. Thus, unless specified otherwise, we refer to the static and temporal network determined by eLSA.

### Cyanobacteria

Our dataset contained 19 cyanobacterial ASVs, which all appeared in the nano-, and nine in the picoplankton. We blasted the sequences against the Cyanorak database [62], v.2. against the nucleotide database containing all *Synechococcus* and *Prochlorococcus* RNAs with the option *e* value 1.0e ^− 5^. We found 2812 sequences comprising 95 different ecotypes (considering name, clade, and subclade), with 93.84–100% identity. A total of 11 BBMO ASVs obtained 63 hits with 100% identity, and within these 63 reference sequences, there were 34 different ecotypes. Most matching sequences were found for *Synechococcus* ASV_1. While *Synechococcus* ASV_5 had only two 100% hits, they did not 100% match ASV_1 (Supplementary Table 5). Finding *Synechococcus* in both size fractions was against expectations, as this genus is part of the pico-plankton. Yet, they have been observed in fractions above 3 µm at BBMO [63]. Recovering *Synechococcus* ASVs from the nanoplankton may be due to cell aggregation, particle attachment, clogging of filters, or being prey to larger microorganisms. *Synechococcus* could be also picked up in the 3-µm filters during cell division.

### Validated associations

As a general rule, the validation of associations tends to be limited as both true interactions and microorganisms that do not interact with each other are poorly known. As done in [48], we determined true genus-genus interactions as those known in the literature, which are compiled within the Protist Interaction Database, PIDA [17]. On October 15th 2019, PIDA contained 2448 interactions. Although our dataset contains protists and bacteria, we could not evaluate bacterial interactions through PIDA, as these are not considered in the database. The ambiguity in taxonomic classification and the large number of edges challenged the validation. We validated associations between microbial eukaryotes via exact string matching as done previously [48].

## Results

### Extracting a temporal network from a single static association network

From 10 years of monthly samples from the Blanes Bay Microbial Observatory (BBMO) in the Mediterranean Sea [30], we computed sequence abundances for 488 bacteria and 1005 microbial eukaryotes from two organismal size-fractions: picoplankton (0.2–3 µm) and nanoplankton (3–20 µm). We removed Archaea since they are not very abundant in the BBMO surface and primers were not optimal to quantify them. We inferred amplicon sequence variants (ASVs) using the 16S and 18S rRNA-gene. After filtering the initial ASV table for sequence abundance and shared taxa among size fractions, we kept 285 and 417 bacterial and 526 and 481 eukaryotic ASVs in the pico- and nanoplankton size fractions, respectively. We found 214 bacterial ASVs that appeared in both size fractions, but only two eukaryotic ASVs: a *Cryothecomonas* (Cercozoa) and a dinoflagellate (Alveolate).

We used 1709 ASVs to infer a preliminary association network with the tool eLSA [46, 47]. Next, we removed environmentally driven edges with EnDED [48]. We only considered edges involving partners that co-occurred more than half of the times together than alone (see the “Methods” section and Fig. 1A-B). Our filtering strategy removed a higher fraction of negative than positive edges (see the “Methods” section and Supplementary Table 1). The resulting network is our single static network connecting 709 nodes via 16,626 edges (16,481 edges, 99.1%, positive and 145, 0.9% negative).

Next, we developed an approach to determine a temporal network. Building upon the single static network, we determined 120 sample-specific (monthly) subnetworks (see the “Methods” section for details). These monthly subnetworks represent the 120 months of the time series and together comprise the temporal network. Each monthly subnetwork contains a subset of the nodes and a subset of the edges of the single static network. We used the ASV abundances indicating the presence (ASV abundance > 0) or absence (ASV abundance = 0) as well as the estimated start and duration of associations inferred with the network construction tool eLSA [46, 47] for determining which nodes and edges are present each month (Fig. 1, see the “Methods” section).

### The single static network metrics differed from most monthly subnetworks

**Fig. 2:**
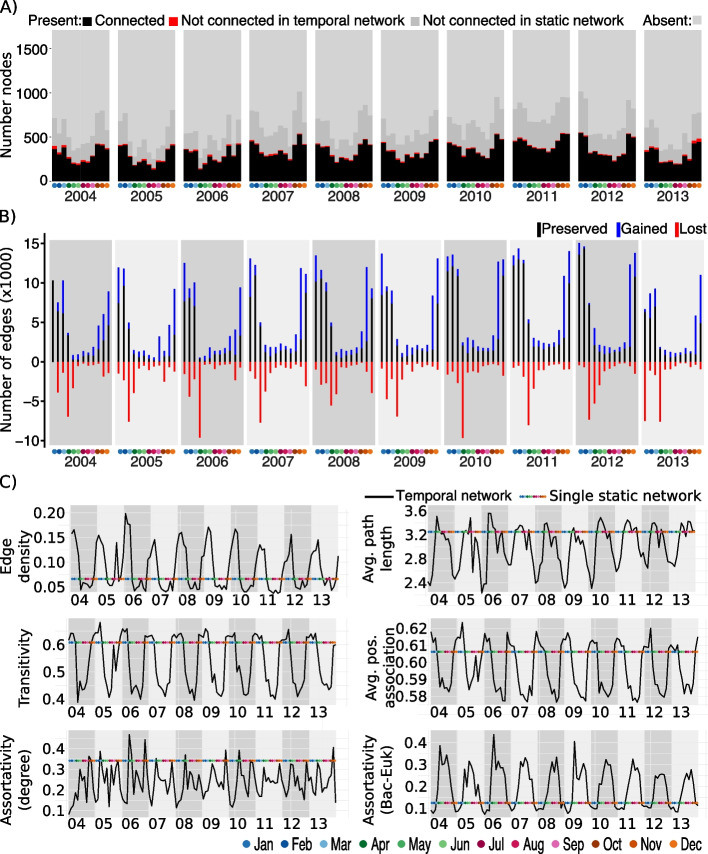
Global (sub)network metrics. **A** Number of ASVs (counting an ASV twice if it appears in both size fractions) for each of the 120 months of the Blanes Bay Microbial Observatory time series. There are 1709 ASVs of which 709 ASVs are connected in the static network. In black, we show the number of nodes connected in the temporal network, and in red, the number of nodes that are isolated in the temporal network, i.e., they are connected in the static network and have a sequence abundance above zero for that month (“non-zero”). In dark gray, we show the number of ASVs that are non-zero in a given month but were not connected in the static and subsequently temporal network. In light gray, we show the number of ASVs with zero-abundance in a given month. The sum of connected and isolated nodes and non-zero ASVs represents each month’s richness (i.e., number of ASVs). **B** By comparing the edges of two consecutive months, i.e., two consecutive monthly subnetworks, we indicate the number of edges that have been lost (red), preserved (black), and those that are gained (blue), compared to the previous month. **C** Six selected global network metrics for each sample-specific subnetwork of the temporal network. The colored line indicates the corresponding metric for the static network

Since each monthly subnetwork was derived from the single static network, they were smaller, containing between 141 (August 2005) and 571 (January 2012) nodes, median ≈354 (Fig. 2A), and between 560 (April 2006) and 15,704 (January 2012) edges, median ≈6052 (Fig. 2B). For further characterization, we computed six global network metrics (Fig. 2C and Methods). The results indicated that the single static network differed from most monthly subnetworks, and it also differed from the average. In general, the single static network was less connected (edge density) and more clustered (transitivity) with higher distances between nodes (average path length) and stronger associations (average positive association score) than most monthly subnetworks (Fig. 2C). In addition, the single static network was usually more assortative according to the node degree but less assortative according to the domain (bacteria vs. eukaryote) than most monthly subnetworks (Fig. 2C). High assortativity indicates that nodes tend to connect to nodes of a similar degree and domain.

### Monthly subnetworks display seasonal behavior with yearly periodicity

Over the analyzed decade, the network became more connected and clustered in colder months, with stronger associations and shorter distances between nodes (Fig. 2C, Supplementary Figures 1 and 2). Most global network metrics indicated seasonal behavior with yearly periodicity (Fig. 2C). For instance, edge density, average positive association score, and transitivity were highest at the beginning and end of each year, while average path length and assortativity (bacteria vs. eukaryotes) were highest in the middle of each year. Assortativity (degree), in contrast to other metrics, usually had two peaks per year corresponding to April–May, and November (Fig. 2C). Some metrics (number of nodes and edges, and average path length) presented similar seasonal behavior with yearly periodicity in the temporal network determined from the single static FlashWeave network (Supplementary Figure 3). However, edge density and transitivity displayed patterns contrary to those observed in the temporal network determined from the single static eLSA network.

We found mainly temperature and day length, and to a lesser extent nutrient concentrations (mainly SiO_2_, NO_3_^*−*^ and NO_2_^*−*^, being PO_4_^3*−*^less relevant), and total chlorophyll-a concentration to affect network topologies as indicated by correlation analyses (Supplementary Figure 2). For example, edge density was highest and temperature lowest in January–March. Then, edge density dropped as temperature increased. April–June displayed edge densities slightly above or similar to those in the warmest months July–September, while October–December had similar or slightly lower edge densities than the coldest months January–March. Edge density vs. hours of light (day length) indicated a yearly recurrent circular pattern for September–April (Supplementary Figure 1). Yet, May–August were not part of the circular pattern. May–August had the highest day length and their corresponding networks low edge density (Supplementary Figure 1).

Next, we quantified how many edges were preserved (kept), lost, and gained (new) in consecutive months. We found the highest loss of edges in April, pointing to a network collapse. The overall number of edges (preserved and gained) was lowest during April–September and increased towards the end of each year (Fig. 2B). The number of associations changed over time in a yearly recurring pattern with few associations being preserved when transitioning from colder to warmer waters. We observed a steep network change when transiting from colder to warmer months, reflecting a large reorganization. In turn, the network change from warmer to colder months was less abrupt. Thus, network change between cold and warm waters was not symmetrical over the studied decade at the BBMO.

We defined summer and winter as in [28] and compared both seasons between consecutive years in terms of preserved, gained, and lost associations and ASVs. We observed higher repeatability in edges (Supplementary Figure 4) and ASVs (results not shown) in colder than in warmer months, indicating higher predictability during low-temperature seasons.

### Potential core associations

A single static network can comprise permanent, seasonal, and temporary associations. By comparing monthly subnetworks, we identified edges that remain (preserved), appear (gained), or disappear (lost) over time (Fig. 2B). Intuitively, we would classify permanent associations through 100% recurrence. However, no association fulfilled the 100% criteria. Most associations had a low recurrence, with three-quarters of the associations present in no more than 38% (total 46) of the monthly subnetworks. The average association prevalence was similar across taxonomic ranks (Supplementary Figure 5). Considering the 100 most prevalent associations, which appeared in 71.7–98.3% (total 86–118) of the monthly subnetworks, 87 were associations among bacteria (Supplementary Table 2).

**Fig. 3:**
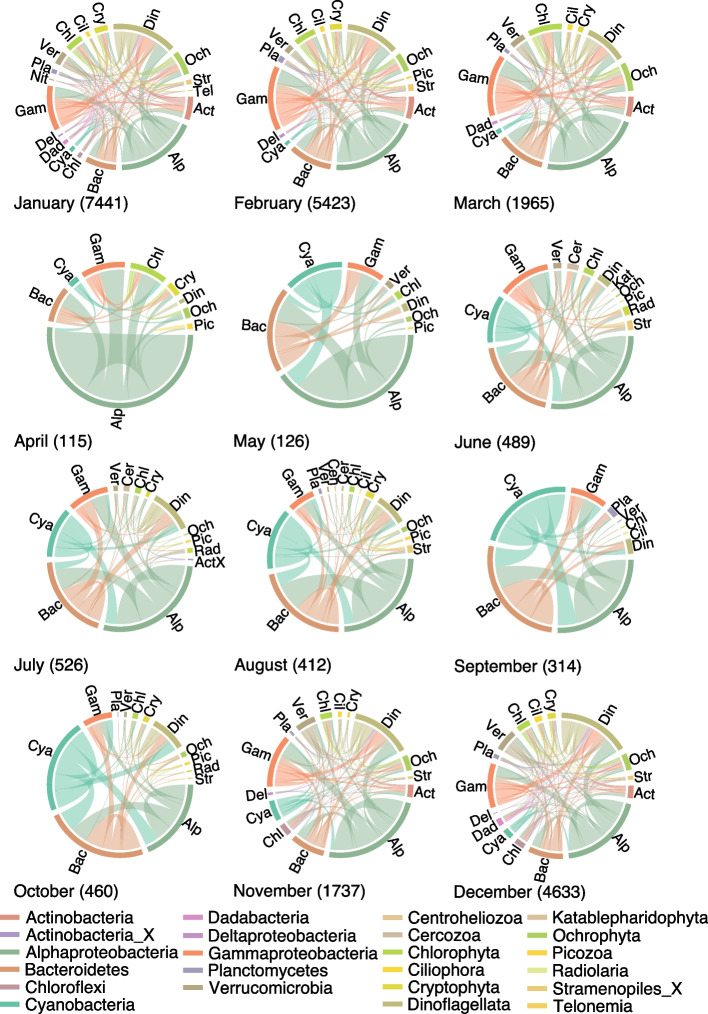
Associations with a monthly prevalence of at least 90%. Bacteria and microbial eukaryotes are separated and ordered alphabetically. We provide in parentheses the number of associations that appeared in at least nine out of ten monthly subnetworks

Although the temporal recurrence of associations over the 10 years was low, we found high recurrence in corresponding months from different years. We quantified the fraction of subnetworks in which each association appeared (Supplementary Figure 6). We observed the highest prevalence from December to March, and the lowest prevalence from June to August (Supplementary Figure 6). For each month, we taxonomically characterized prevalent associations appearing in at least nine out of the ten monthly subnetworks (e.g., 9 out of 10 Januarys; Fig. 3). We found a larger number of prevalent associations in colder waters compared to warmer waters, with Alphaproteobacteria dominating these associations, especially in April and May (Fig. 3). The Alphaproteobacteria ASVs featuring highly prevalent associations belonged to *Pelagibacter ubique* (SAR11 Clades Ia & II), Rhodobacteraceae, *Amylibacter*, Puniceispirillales (SAR116), *Ascidiaceihabitans*, *Planktomarina*, Parvibaculales (OCS116), and *Kiloniella*. Between April and May, we noticed a large increase in the fraction of associations including Cyanobacteria or Bacteroidetes as association partners. While Cyanobacteria associations were a small fraction during November–April, they had a dominant role from May to October along with Bacteroidetes and Alphaproteobacteria associations (Fig. 3). Overall, this underlines the dynamic nature of associations over the year, pointing to recurring annual associations that may be essential for ecosystem function.

### Dynamic associations within main taxonomic groups: the case of Cyanobacteria

**Fig. 4:**
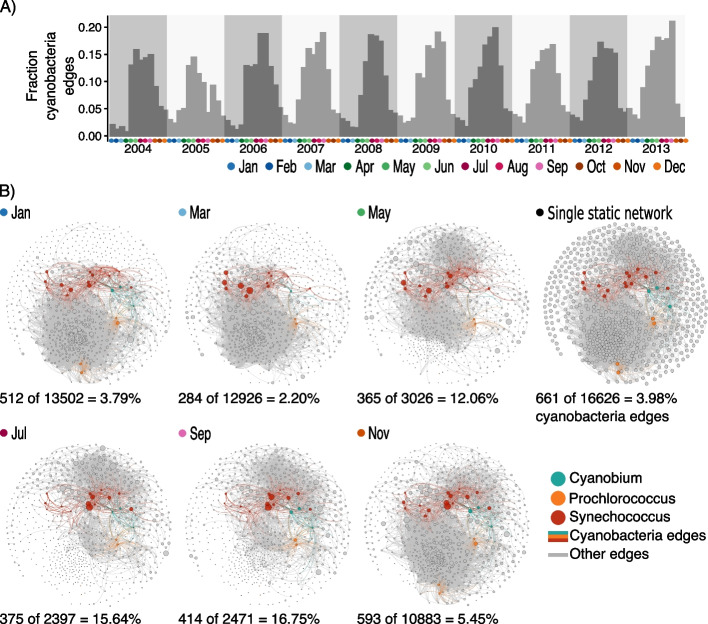
*Cyanobacteria* associations. **A** Fraction of edges in the temporal network containing at least one *Cyanobacteria* ASV. **B** Location of *Cyanobacteria* associations in the temporal network and the single static network. Here, we show, as an example, selected months of year 2011. The number and fraction of cyanobacterial edges and total number of edges are listed below each monthly subnetwork and the single static network

Our results indicated that associations are dynamic within specific taxonomic groups. Therefore, we investigated their behavior in Cyanobacteria given the importance of this group as primary producers in the ocean. We found 661 associations for *Synechococcus*, *Prochlorococcus*, and *Cyanobium* ASVs (Fig. 4 and Supplementary Figure 7). Most associations between cyanobacterial ASVs were positive (63 of 65), and only a *Synechococcus* (referred to as bn_ASV_5) was negatively associated (association score − 0.5) with other *Synechococcus* (bn_ASV_1 and bn_ASV_25), which, in turn, were positively associated (association score 0.8). While bn_ASV_5 appeared mainly in colder months, the other two appeared mainly in warmer months (Supplementary Figure 7). All Cyanobacteria had more associations with other bacteria (in total 433) than with eukaryotes (in total 163).

Within the temporal network, the fraction of Cyanobacteria associations was highest in April–October (Fig. 4A), which are the months with the largest cyanobacterial abundances (Supplementary Figure 7), and the fewest edges in the entire temporal network (Fig. 2B), for example, in the year 2011 (Fig. 4B). We found that cyanobacterial ASVs, although being evolutionarily related, behaved differently in terms of the number of associations over time, and association partners (Supplementary Figure 7). For example, *Synechococcus* bn_ASV_5 had fewer partners than bn_ASV_1 according to numbers of associations, but more according to taxonomic variety (Supplementary Figure 7). Only a tiny fraction of *Prochlorococcus* (e.g., bp_ASV_18) association partners were other Cyanobacteria, which contrasted with *Synechococcus* and *Cyanobium* (Supplementary Figure 7). Moreover, we observed that *Cyanobium* (bn_ASV_20) connected to one Deltaproteobacteria (SAR324) ASV during the first 8 years, but the association disappeared in the last 2 years. In particular, the inferred association duration was 101 months, starting in March 2004 and ending in July 2012. After summer 2012, the Deltaproteobacteria ASV was not detected except a few reads in November and December 2012 and 2013. This Cyanobacteria example may also illustrate the dynamics of associations within other main taxonomic groups.

### Validating associations using known ecological interactions

We checked how many potential interactions could be validated using a database of observed ecological interactions (PIDA; [17]). In total, 16 associations (out of 16,626) in the temporal network were validated by PIDA (Supplementary Table 6). These 16 associations describe six unique interactions between seven taxa (at the genus level). For instance, the reoccurring association between a diatom from genus *Thalassiosira* and a Flavobacteriia starts mainly around October and often ends around March (Supplementary Figure 8). In contrast, the reoccurring association between a dinoflagellate from genus *Gyrodinium* and one from *Heterocapsa* appears for a shorter time and during the summer months (Supplementary Figure 8).

## Discussion

Previous work identified yearly recurrence of microbial community composition at the BBMO [13, 28, 64], and similarly at the nearby Bay of Banyuls [14], both in the North-West Mediterranean Sea and in other temperate sites around the world [12, 65]. We focused here in the connectivity of microorganisms, and how they organize themselves from a network perspective. In general, the measured global network metrics (edge density, transitivity, and average path length) are within the range reported in previous studies [22–25, 66–68] (Table 2). Contrary to early studies reporting biological networks generally being disassortative (negative assortativity based on degree) [69], our single static network and the monthly subnetworks were assortative. Microorganisms had more and stronger connections and a tighter clustering in colder than in warmer waters. To some extent, this might reflect species richness, which has been shown for the resident microorganisms to increase during the colder months at BBMO using the same dataset [28]. However, the exact effect of richness on ecological interactions among microorganisms needs further analysis. Seasonal bacterial freshwater networks [67] also showed higher clustering in fall and winter than in spring and summer, but, in contrast to our results, networks were most extensive in summer and smallest in winter. In agreement with our results, Chaffron et al. [68] reported higher association strength, edge density, and transitivity in cold polar regions compared to other warmer regions of the global ocean. Cold waters in the Mediterranean Sea are milder than polar waters. However, together, these results suggest that either microorganisms interact more in colder environments or that their recurrence is higher due to higher environmental selection exerted by low temperatures. Additionally, limited resources (mainly nutrients) in summer or in the tropical and subtropical open ocean may prevent the establishment of several microbial interactions. In any case, temperature is likely not the only driver of network architecture [68].

**Table 2:** Global network metrics of previously described microbial association networks

Location and depth	Edge density	Transitivity	Average path length	Sampling	Domains	Notes	Reference
SPOT (off the southern California coast); deep chlorophyll maximum	0.04	0.26	3.05	Monthly. August 2000–March 2004	Archaea, bacteria, and eukaryotes	Edge density for microbial network including environmental factors. Transitivity and average path length for microbial network	[22]
SPOT; surface ocean and deep chlorophyll maximum	0.14	0.33	1.94	Monthly. August 2000–January 2011	Free-living bacteria and picoeukaryotes	Metrics from surface layer network	[23]
SPOT; surface	0.02	0.24		Monthly. March 2008–January 2011	Free-living eukaryotes (0.7–20 µm), bacteria (0.22–1 µm) and viruses (30 kDa–0.22 µm)		[24]
SPOT; five depths (5 m—surface, the deep chlorophyll maximum layer, 150 m, 500 m and 890 m—just above the sea floor)	0.04	0.28	2.07	Monthly. August 2003–January 2011	Free-living bacteria	Metrics for a 5-m layer network	[25]
52 samples from freshwater lakes in China; surface	(0.023) W:0.033, Sp:0.032, S:0.036, F:0.029	(0.472) W:0.518, Sp:0.480, S:0.475, F:0.573	(4.84) W:2.16, Sp:5.03 S:7.26, F:3.04	Spatial	Bacteria	Metrics for (whole network) and seasonal networks: W: winter, Sp: spring, S: summer, and F: fall	[67]
68 stations from the Tara Oceans expedition across eight oceanic provinces; surface and deep chlorophyll maximum	0.005, 0.003, 0.008	0.2, 0.0, 0.43	3.05, 3.02, 2.56	Spatial	Organisms from seven size fractions	Metrics from surface networks including eukaryotes only, eukaryotes and prokaryotes (0.5–5 µm), and prokaryotes only (0.2–1.6 µm) respectively	[66]
115 stations from the Tara Oceans expedition covering all major oceanic provinces from pole to pole; surface and deep chlorophyll maximum	0.002	0.036		Spatial	Bacteria, archaea, and eukaryotes from six size fractions	Metrics represent the means of sample-specific subnetworks	[68]

The effects of environmental variables on network metrics are unclear [70], yet, our approach allowed us to identify potential environmental drivers of network architecture. Correlation analyses pointed to variables that have been found to influence microbial abundances in the ocean. For instance, our results indicated that temperature and day length, key variables driving microbial assemblages in seasonal time series [12–14], and to a lesser extent inorganic nutrients, were the main factors influencing global network metrics. It also agrees with earlier works indicating that phosphorus and nitrogen are the primary limiting nutrients in the Western Mediterranean Sea [71, 72]. Altogether, our correlation analysis is a step forward towards elucidating the effects of environmental variables on network metrics. However, we did not consider several other variables that could affect network architecture (e.g., organic matter).

Our preliminary network (significant associations derived with eLSA) contained 18% negative edges compared to 0.9% in the single static network (after filtering). Thus, our filtering strategy removed proportionally more negative edges. Associations may represent positive or negative interactions, but they can also indicate high niche overlap (positive association) or divergent niches (negative association) between microorganisms [73]. We hypothesize that most of the removed negative edges represented associations between microorganisms from divergent niches, most likely corresponding to colder or warmer months.

We found more highly prevalent associations within specific months than when considering all 10 years of data. Furthermore, our results indicate a potentially low number of core interactions and a vast number of non-core ones. Usually, core microorganisms are defined based on sequence abundances, as those microorganisms (or taxonomical groups) appearing in all samples or habitats being under investigation [74]. Shade and Handelsman [74] suggested that other parameters, including connectivity, should create a more complex portrait of the core microbiome and advance our understanding of the role of key microorganisms and functions within and across ecosystems [74]. Using a temporal network, we identified core associations based on recurrence, which contributes to our understanding of key interactions underpinning microbial ecosystem functions. Considering associations within each month, we found more highly prevalent associations in colder than in warmer months. Our results indicate microbial connectivity is more repeatable (indicating higher predictability) in colder than in warmer waters. On the one hand, the microbial community in colder waters being more recurrent [13] may explain our observations indicating a more robust connectivity during this period. Alternatively, it may be the stronger connectivity what leads to more similar communities in colder waters at the BBMO. Last but not least, the interplay of both species dynamics and interactions may determine community turnover in the studied ecosystem. From a technical viewpoint, our monthly sampling strategy and/or the overall single static network may have not been able to detect interactions appearing solely in summer resulting in smaller monthly subnetworks. For instance, previous work on freshwater lakes constructed season-specific networks and found more associations in summer than in winter [67].

Several network-based analyses have been used to particularly study Cyanobacteria associations. For example, in the southern Californian coast, Chow et al. [24] identified 44 potential relationships of 12 Cyanobacteria (*Prochlorococcus* and *Synechococcus*) with two potential eukaryote grazers (a ciliate and a dinoflagellate), 39 to other bacteria, and three between Cyanobacteria, which were all positive. Similarly, all cyanobacterial ASVs in our study connected primarily to other bacterial ASVs and featured mainly positive associations. Furthermore, Cyanobacteria displayed primarily positive associations with other microorganisms in a global ocean network [66]. This suggests that other sampling or computational approaches are needed to detect negative associations involving marine cyanobacteria.

Identifying different potential association partners for closely related Cyanobacteria may indicate adaptations to different niches. A recent study found distinct seasonal patterns for closely related bacterial taxa indicating niche partitioning at the BBMO, including *Synechococcus* ASVs [64]. Our approach can complement and further characterize “sub-niches” by providing association partners for different ASVs. Moreover, in contrast to a single static network, temporal networks allow identifying associated partners in time (Supplementary Figure 7). An increase in the abundance of a microorganism may promote the growth of associated partners and a decrease may hinder the growth of partners or cause predators to prey on other microorganisms. Moreover, given the majority of association partners being other bacteria, the growth of Cyanobacteria may affect other bacteria and their growth, which is why it is necessary to identify potential interaction partners [67].

Our approach allowed us to disentangle in time the associations captured by a single static network using monthly samples for ten years. Future studies should determine whether higher sampling frequency (e.g., daily samples during a month) can capture other associations not present in our networks. Thus, our results should be considered taking into account the used (monthly) sampling frequency. In addition, certain network metrics may depend on the tool used to infer the single static network, e.g., edge density, and, therefore, should be interpreted with care. An additional consideration is that we disregarded local network patterns by using global network metrics. Future work could use the local-topological metric based on graphlets [75]. Counting the number of graphlets a node is part of quantifies their local connection patterns, which allows inferring seasonal microorganisms through recurring connection patterns in a temporal network.

## Conclusion

Incorporating the temporal dimension in microbial association analysis unveiled multiple patterns that often remain hidden when using single static networks. Investigating a coastal marine microbial ecosystem over 10 years revealed a 1-year-periodicity in the network topology. The temporal network architecture was not stochastic, but displayed a modest amount of recurrence over time, especially in winter. Future efforts to understand the ocean microbiome should consider the dynamics of microbial interactions as these are likely fundamental for ecosystem functioning.

### Supplementary Information

**Additional file 1: Supplementary Figure 1.** Correlation analysis. Using the temporal network, we correlated six global network metrics with environmental factors including the nutrients PO_4_^3−^, NH_4_^+^, NO_2_^−^, NO_3_^−^, and SiO_2_. The global network metrics were: Edge density, Average positive association (Avg. pos. ass.) score, Transitivity, Average path length (Avg. path length), Assortativity (degree), and Assortativity (bacteria vs. eukaryote). Each dot is a sample-specific subnetwork and its color indicates the month it represents. Also, the linear regression line with a 0.95 confidence interval is shown in grey.

**Additional file 2: Supplementary Figure 2.** Correlation analysis through linear regression. Using the temporal network, we correlated six global network metrics with environmental factors including the nutrients PO_4_^3−^, NH_4_^+^, NO_2_^−^, NO_3_^−^, and SiO_2_. The global network metrics were: Edge density, Average positive association (Avg. pos. ass.) score, Transitivity, Average path length (Avg. path length), Assortativity (degree), and Assortativity (bacteria vs. eukaryote). The number, circle's size, and color in the square correspond to the Spearman correlation scores, no circle indicates non-significance.

**Additional file 3: Supplementary Figure 3.** Global (sub)network metrics. Number of nodes, number of edges, and six selected global network metrics for each sample-specific subnetwork of the temporal network determined with FlashWeave.

**Additional file 4: Supplementary Figure 4.** Number of preserved, gained, and lost edges in summer and winter. A) Indicates how we determined summer, shown with red dots (temperature above 17 ºC and day length above 14 hours) and winter, shown with blue dots (temperature below 17 ºC and day length below 11 hours); grey dots indicate months that are neither summer nor winter. B) Accumulation curve of ASVs per year for winter (blue) and summer (red). C) and D) number of preserved, gained, and lost edges for winter and summer, respectively. The colors of flows indicate the prevalence of an edge with 10 (light blue) being present in each year, and 1 (dark blue) appearing in only one year. An edge appears in a year if it appears in at least one monthly subnetwork in the corresponding season. In winter, most edges appear in all years (light blue indicating 100% prevalence with edges present in all ten years), i.e., most edges are preserved in the consecutive months (we see a flow from the blue preserved box to the next blue box). In summer, compared to winter, fewer edges are present in a month (combination of boxes indicating preserved, first time gained, and gained), and more edges are (re)gained and lost throughout the years (subsequently, prevalence is lower indicated through darker blue).

**Additional file 5: Supplementary Figure 5.** Association prevalence increases slightly when microorganisms are taxonomically more related. We grouped the associations according to the taxonomic classification of association partners (columns) and size fractions (rows). For example “Class” groups associations between bacteria and eukaryotes, respectively, which were assigned to the same class. The grey column groups associations between bacteria and eukaryotes. The boxplot shows the association prevalence over a decade, i.e., in how many monthly subnetworks an association appears (given as a fraction from 0 to 100% = 120 networks).

**Additional file 6: Supplementary Figure 6.** Association prevalence per month. Big bar plots: distribution of associations' prevalence for each month. For example, the bar at 100 for January indicates the number of edges that have been present in all Januarys of the ten-year time series. Small bar plots: number of nodes forming the associations with a 100% prevalence. For example, only bacteria were responsible for the edges during May, with an association prevalence of 100%. Bacteria are indicated with B or b, eukaryote with E or e. ASVs from the nano size-fraction have a capital letter (B, E), and ASVs from the pico size-fraction have a small letter (b, e).

**Additional file 7: Supplementary Figure 7.** Association partners of Cyanobacteria. The number of Cyanobacteria associations in the temporal network (stacked bars) and the cyanobacterial sequence abundance in each month (black dashed line). Within the box, figures are split by ASVs (rows) and size fractions: picoplankton (left column) and nanoplankton (right column). The unboxed plots on the right are ASVs detected only in the nanoplankton. The height of the bar indicates the number of edges in each month for each cyanobacterial ASV. The color indicates the taxonomy of the association partner. From bottom to top, first appear bacteria, and then eukaryotes, both sorted alphabetically. The subtitle shows the number of association partners followed by an identifier (first 3 letters) for bacteria and eukaryotes.

**Additional file 8: Supplementary Figure 8.** Microbial association partners that have been reported in the literature. Found associations in the temporal network (one association per panel and a black dot on the bottom shows presence in the monthly subnetwork) and the sequence abundance in each month (solid and dashed lines). The color and line type indicate the taxonomy of the association partners.

**Additional file 9: Supplementary Table 1.** Number of nodes, removed isolated nodes, and number and fraction of edges in the preliminary network (A), and network obtained after removing environmentally-driven edges (B) and edges with association partners appearing more often alone than with the partner (C), which is the single static network. For comparison, we also give the minimum and maximum number of nodes and edges for the temporal network (D). We did not determine the union and intersection for the temporal network. If an ASV appeared in the nano and pico size fraction, it is counted twice. Therefore, for A-C) we also determined the number of microorganisms not considering size fraction (union) and being present in both size fractions (both, i.e., intersection).

**Additional file 10: Supplementary Table 2.** Top 100 most prevalent/recurring associations.

**Additional file 11: Supplementary Table 3.** Number of environmental factors leading to the removal of edges.

**Additional file 12: Supplementary Table 4.** Number of environmentally-driven edges for each environmental factor and fraction considering the total number of edges (29820) in the network. In addition, we present the number of positive and negative edges and the fraction considering the number of edges removed through an environmental factor.

**Additional file 13: Supplementary Table 5.** 100% Matching sequences from Cyanorak database for selected cyanobacterial ASVs.

**Additional file 14: Supplementary Table 6.** Interactions found in the BBMO temporal network that have been reported in the literature. The table shows the number of associations found in the network. For example, the association between the ASVs classified as Dia. *Thalassiosira* and ASVs classified as F. unknown Flavobacteriia has been found 6 times in the network.

## Data Availability

The BBMO microbial sequence abundances (ASV tables), taxonomic classifications, environmental data including nutrients, networks, and R-Markdowns for the data analysis including commands to run eLSA and EnDED (environmentally driven-edge-detection and computing Jaccard index) are publicly available: https://github.com/InaMariaDeutschmann/TemporalNetworkBBMO. DNA sequences are publicly available at the European Nucleotide Archive (https://www.ebi.ac.uk/ena; accession numbers PRJEB23788 for 18S rRNA genes & PRJEB38773 for 16S rRNA genes).
