# Supplementary figures and images for: Disentangling temporal associations in marine microbial networks
Source: Microbiome. 2023 Apr 21;11:83. doi: 10.1186/s40168-023-01523-z (PMC10120119; doi:10.1186/s40168-023-01523-z)

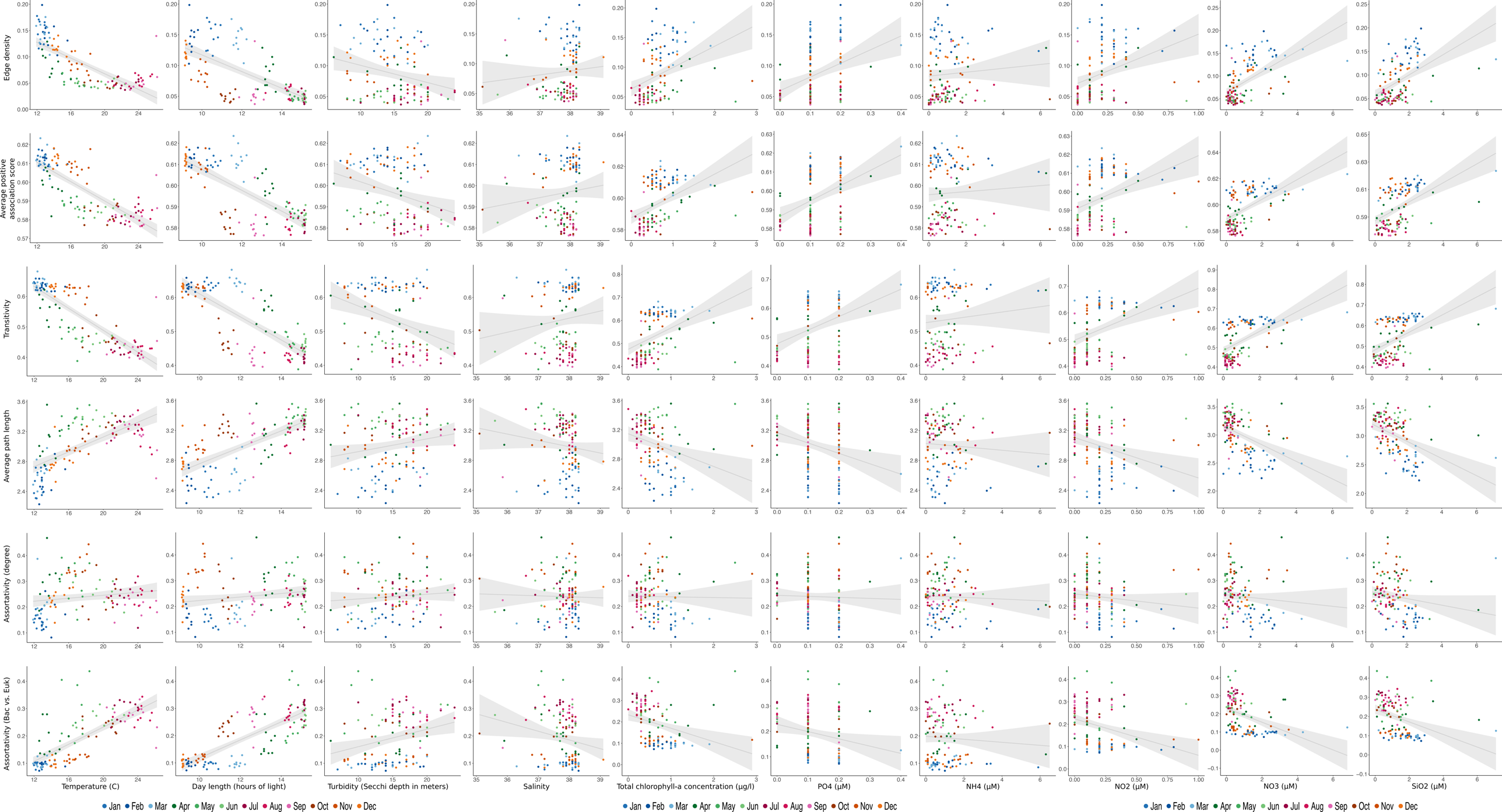

Supplement: Supplementary file 2 — Additional file 1: Supplementary Figure 1. Correlation analysis. Using the temporal network, we correlated six global network metrics with environmental factors including the nutrients PO43−, NH4+, NO2−, NO3−, and SiO2. The global network metrics were: Edge density, Average positive association (Avg. pos. ass.) score, Transitivity, Average path length (Avg. path length), Assortativity (degree), and Assortativity (bacteria vs. eukaryote). Each dot is a sample-specific subnetwork and its color indicates the month it represents. Also, the linear regression line with a 0.95 confidence interval is shown in grey. [file 40168_2023_1523_MOESM1_ESM.pdf]

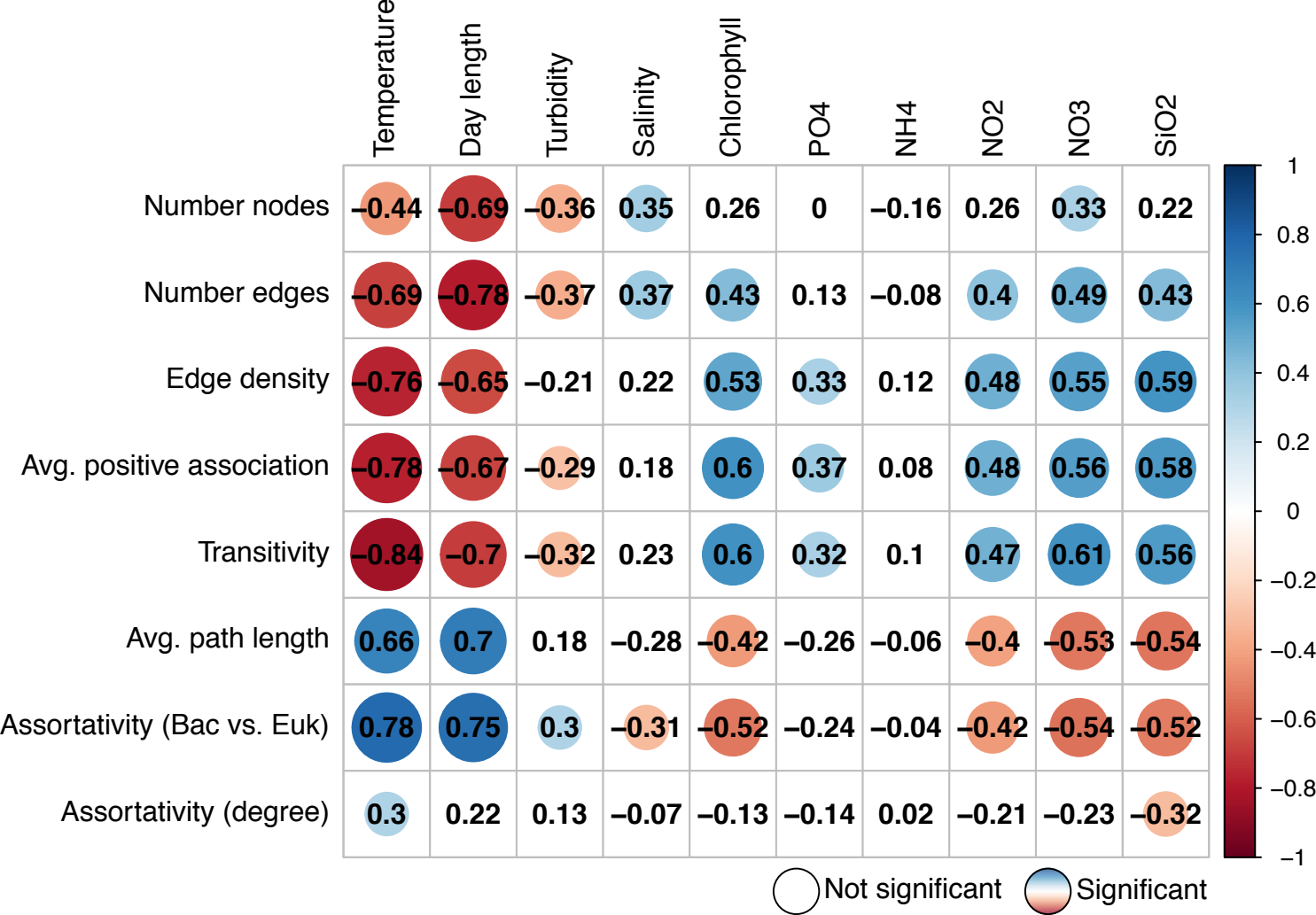

Supplement: Supplementary file 3 — Additional file 2: Supplementary Figure 2. Correlation analysis through linear regression. Using the temporal network, we correlated six global network metrics with environmental factors including the nutrients PO43−, NH4+, NO2−, NO3−, and SiO2. The global network metrics were: Edge density, Average positive association (Avg. pos. ass.) score, Transitivity, Average path length (Avg. path length), Assortativity (degree), and Assortativity (bacteria vs. eukaryote). The number, circle's size, and color in the square correspond to the Spearman correlation scores, no circle indicates non-significance. [file 40168_2023_1523_MOESM2_ESM.pdf]

# FlashWeave with environmental factors

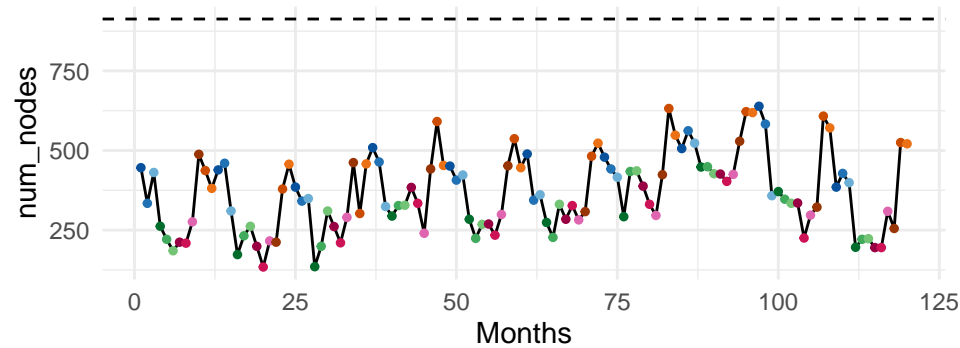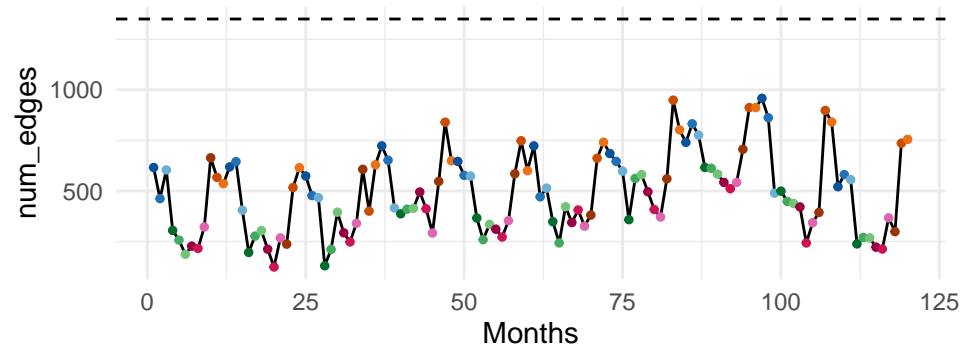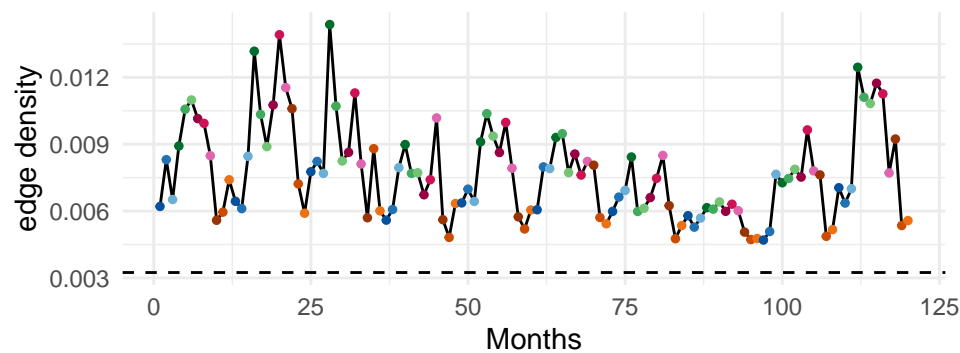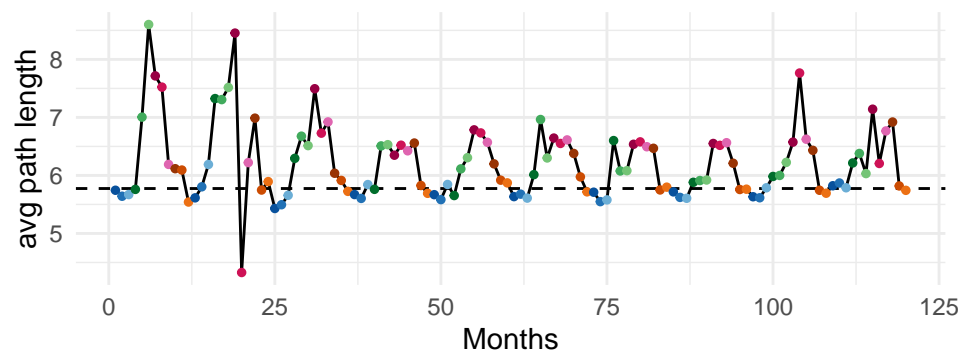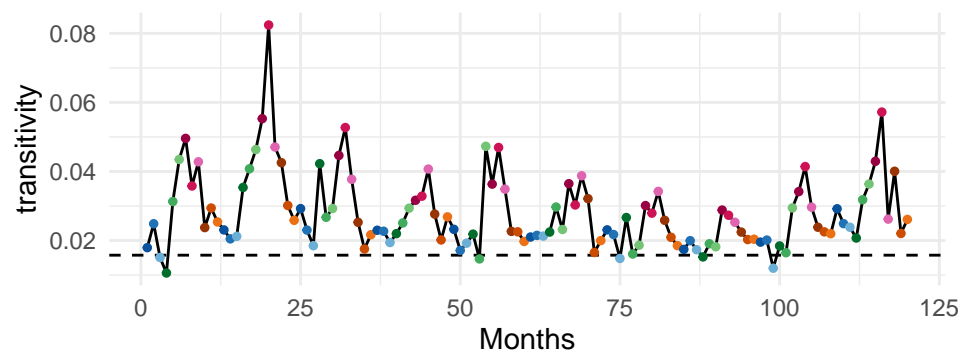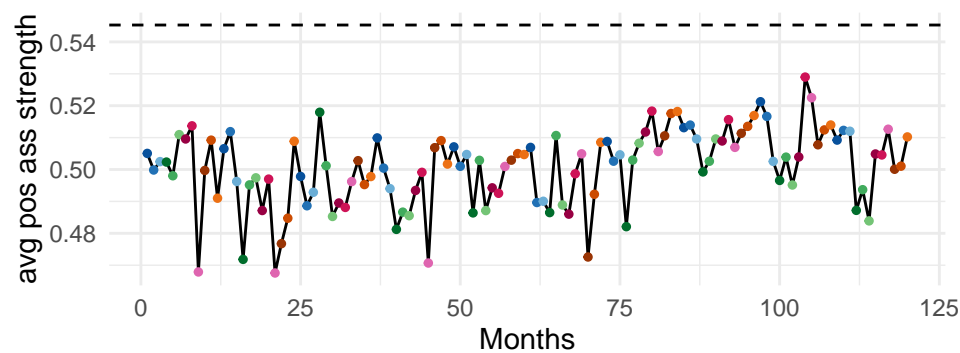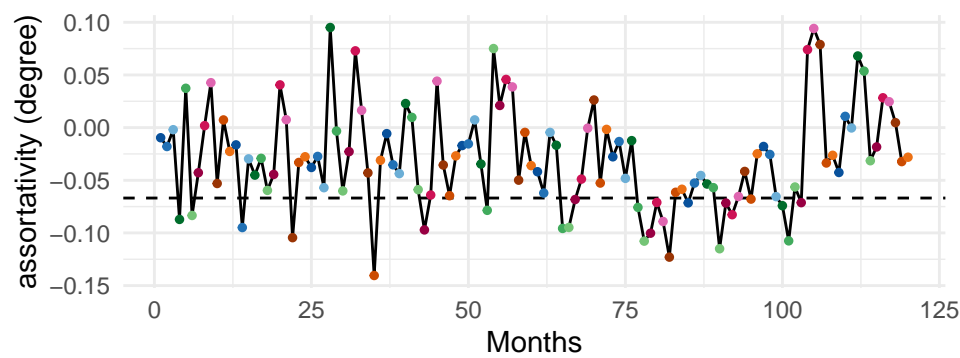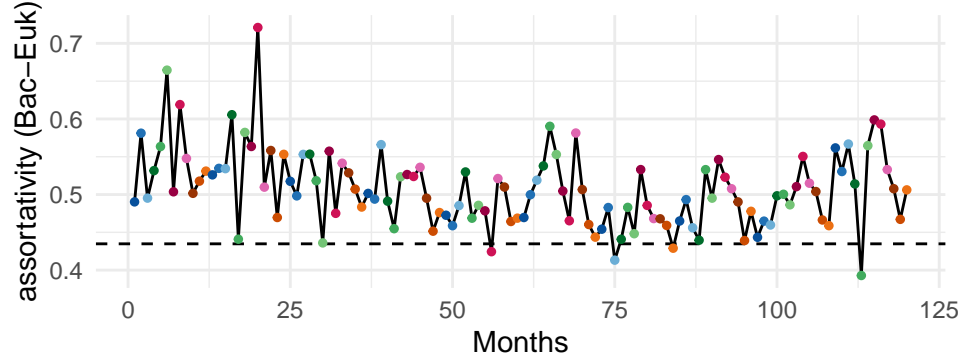

Supplement: Supplementary file 4 — Additional file 3: Supplementary Figure 3. Global (sub)network metrics. Number of nodes, number of edges, and six selected global network metrics for each sample-specific subnetwork of the temporal network determined with FlashWeave. [file 40168_2023_1523_MOESM3_ESM.pdf]

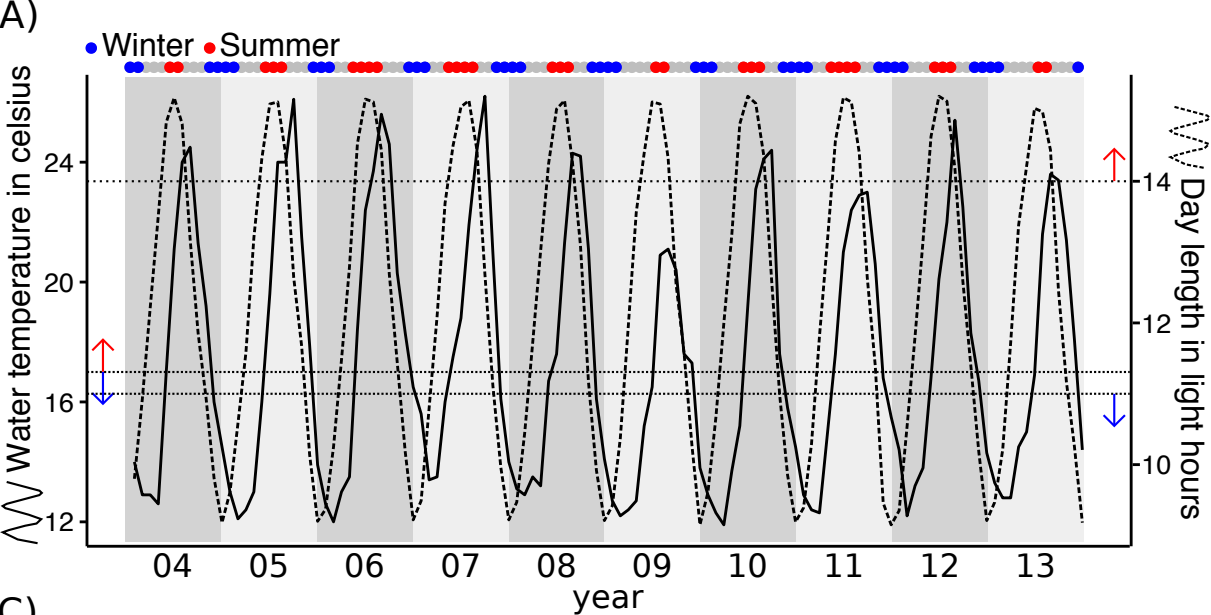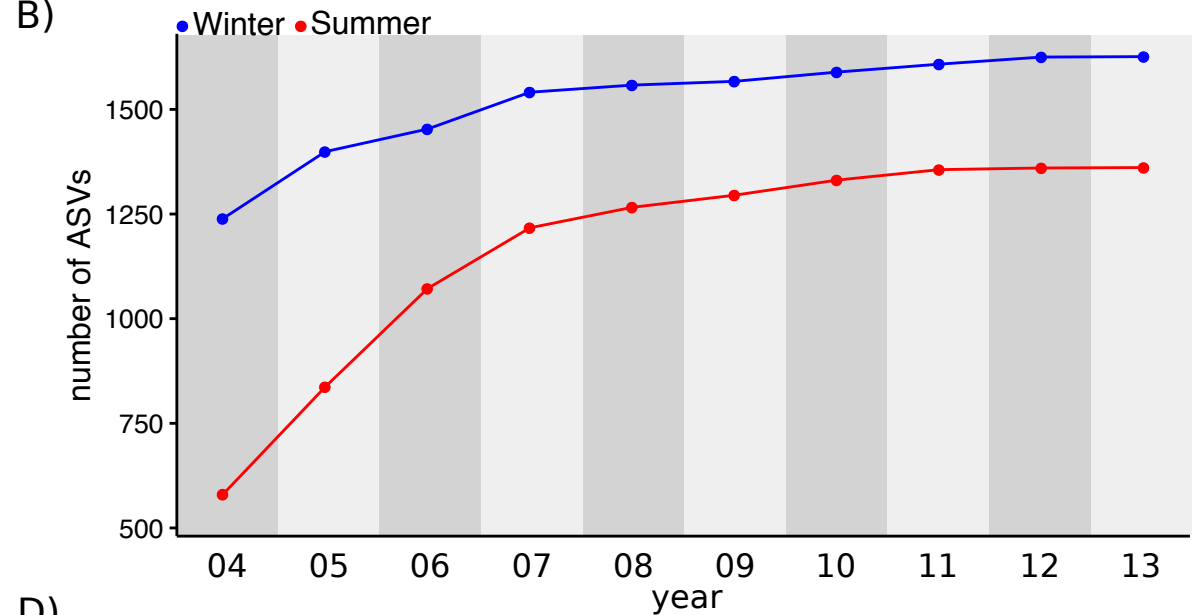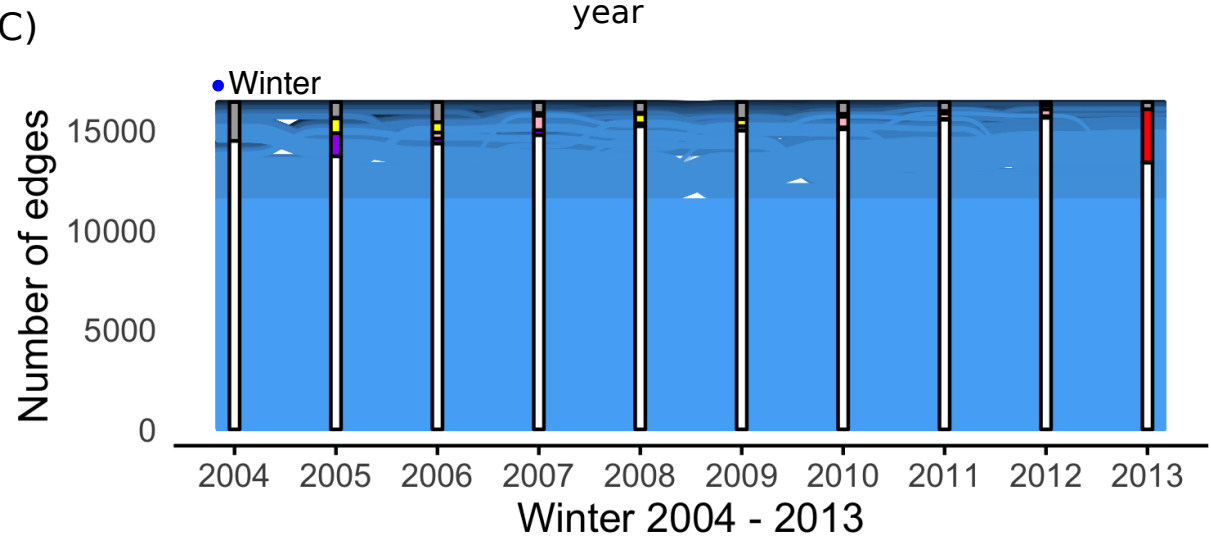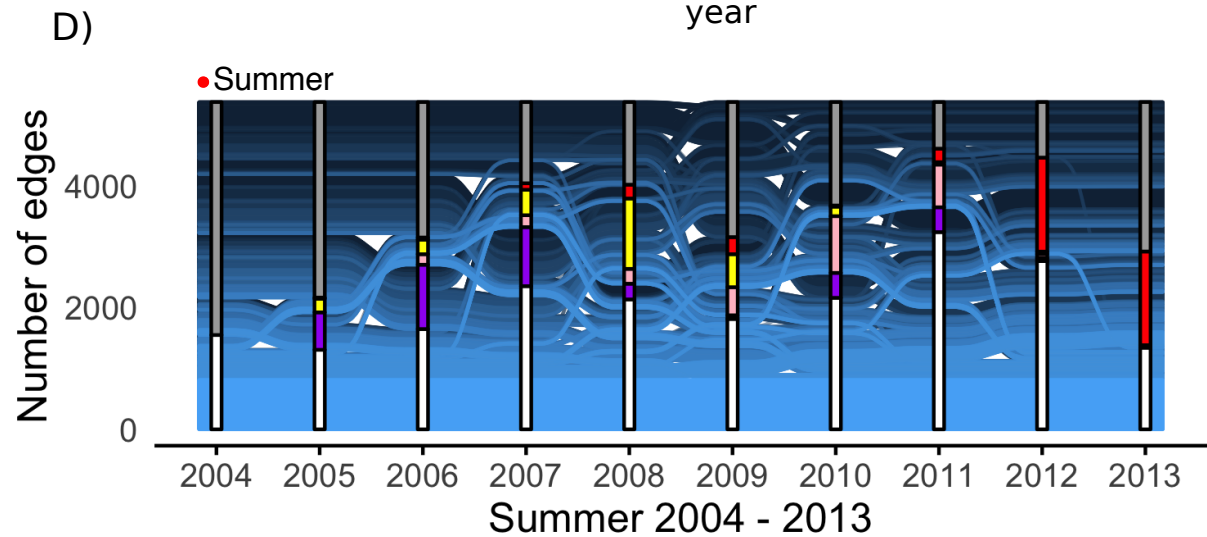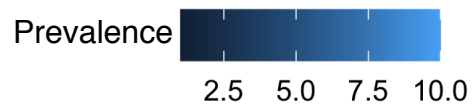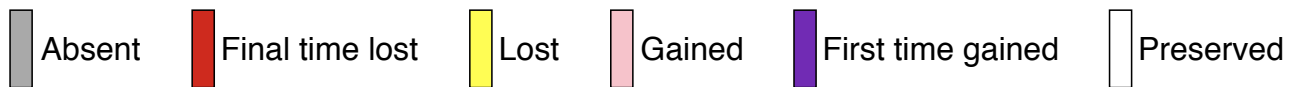

Supplement: Supplementary file 5 — Additional file 4: Supplementary Figure 4. Number of preserved, gained, and lost edges in summer and winter. A) Indicates how we determined summer, shown with red dots (temperature above 17 ºC and day length above 14 hours) and winter, shown with blue dots (temperature below 17 ºC and day length below 11 hours); grey dots indicate months that are neither summer nor winter. B) Accumulation curve of ASVs per year for winter (blue) and summer (red). C) and D) number of preserved, gained, and lost edges for winter and summer, respectively. The colors of flows indicate the prevalence of an edge with 10 (light blue) being present in each year, and 1 (dark blue) appearing in only one year. An edge appears in a year if it appears in at least one monthly subnetwork in the corresponding season. In winter, most edges appear in all years (light blue indicating 100% prevalence with edges present in all ten years), i.e., most edges are preserved in the consecutive months (we see a flow from the blue preserved box to the next blue box). In summer, compared to winter, fewer edges are present in a month (combination of boxes indicating preserved, first time gained, and gained), and more edges are (re)gained and lost throughout the years (subsequently, prevalence is lower indicated through darker blue). [file 40168_2023_1523_MOESM4_ESM.pdf]

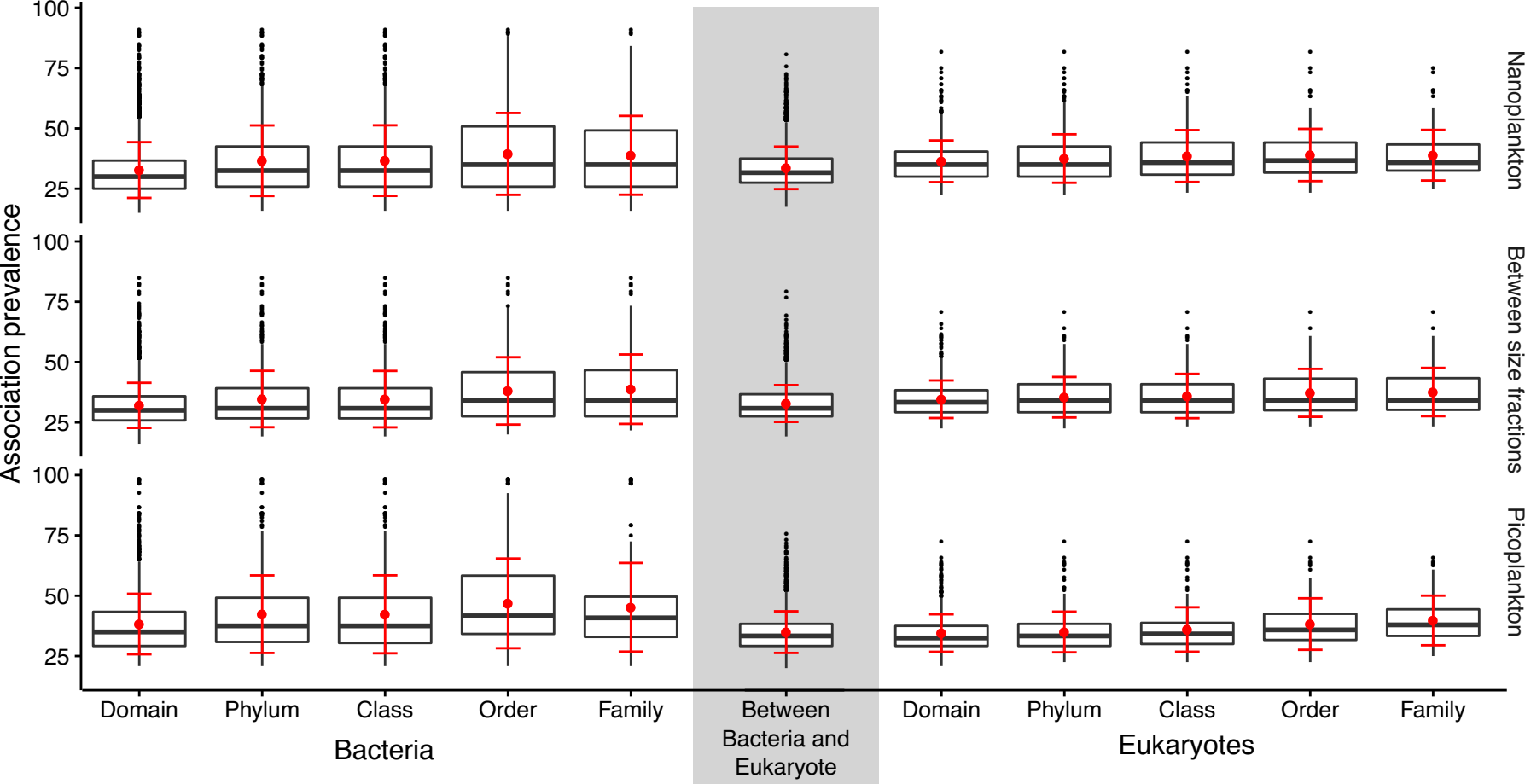

Supplement: Supplementary file 6 — Additional file 5: Supplementary Figure 5. Association prevalence increases slightly when microorganisms are taxonomically more related. We grouped the associations according to the taxonomic classification of association partners (columns) and size fractions (rows). For example “Class” groups associations between bacteria and eukaryotes, respectively, which were assigned to the same class. The grey column groups associations between bacteria and eukaryotes. The boxplot shows the association prevalence over a decade, i.e., in how many monthly subnetworks an association appears (given as a fraction from 0 to 100% = 120 networks). [file 40168_2023_1523_MOESM5_ESM.pdf]

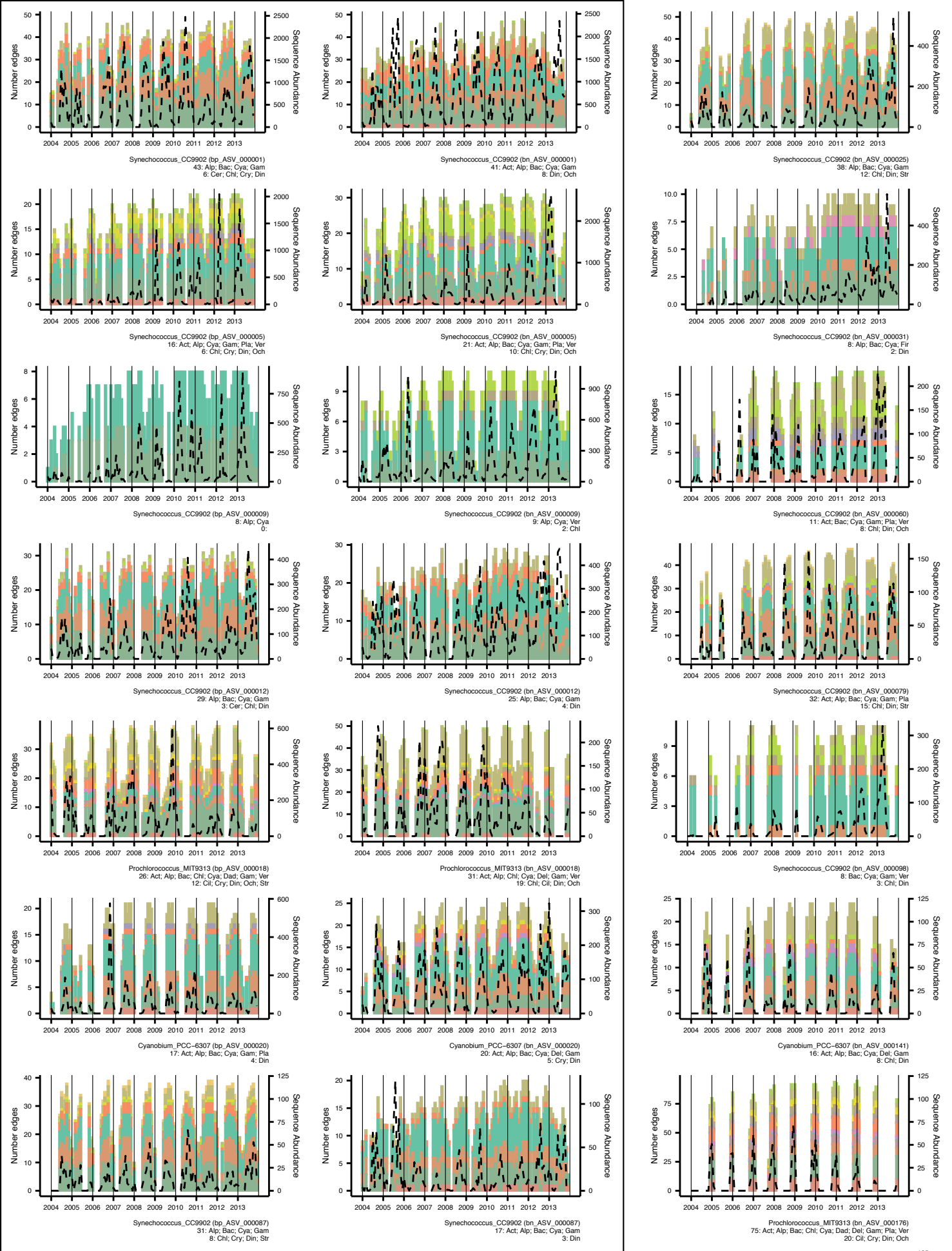

Supplement: Supplementary file 8 — Additional file 7: Supplementary Figure 7. Association partners of Cyanobacteria. The number of Cyanobacteria associations in the temporal network (stacked bars) and the cyanobacterial sequence abundance in each month (black dashed line). Within the box, figures are split by ASVs (rows) and size fractions: picoplankton (left column) and nanoplankton (right column). The unboxed plots on the right are ASVs detected only in the nanoplankton. The height of the bar indicates the number of edges in each month for each cyanobacterial ASV. The color indicates the taxonomy of the association partner. From bottom to top, first appear bacteria, and then eukaryotes, both sorted alphabetically. The subtitle shows the number of association partners followed by an identifier (first 3 letters) for bacteria and eukaryotes. [file 40168_2023_1523_MOESM7_ESM.pdf]

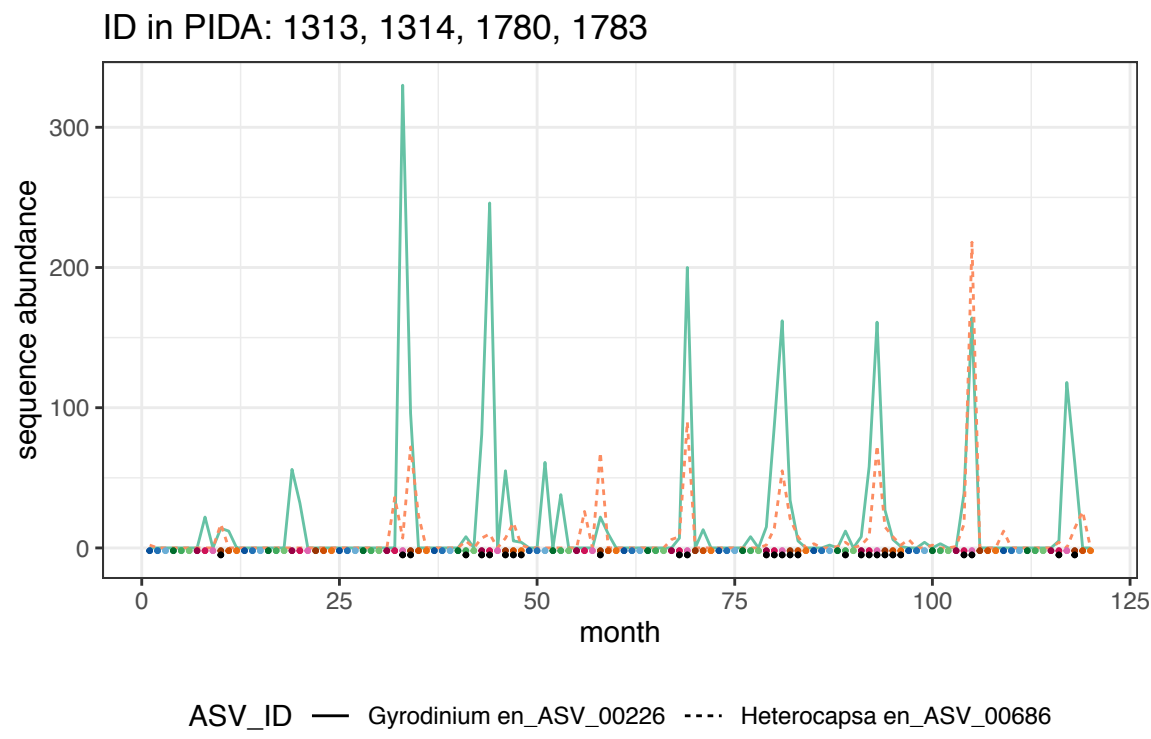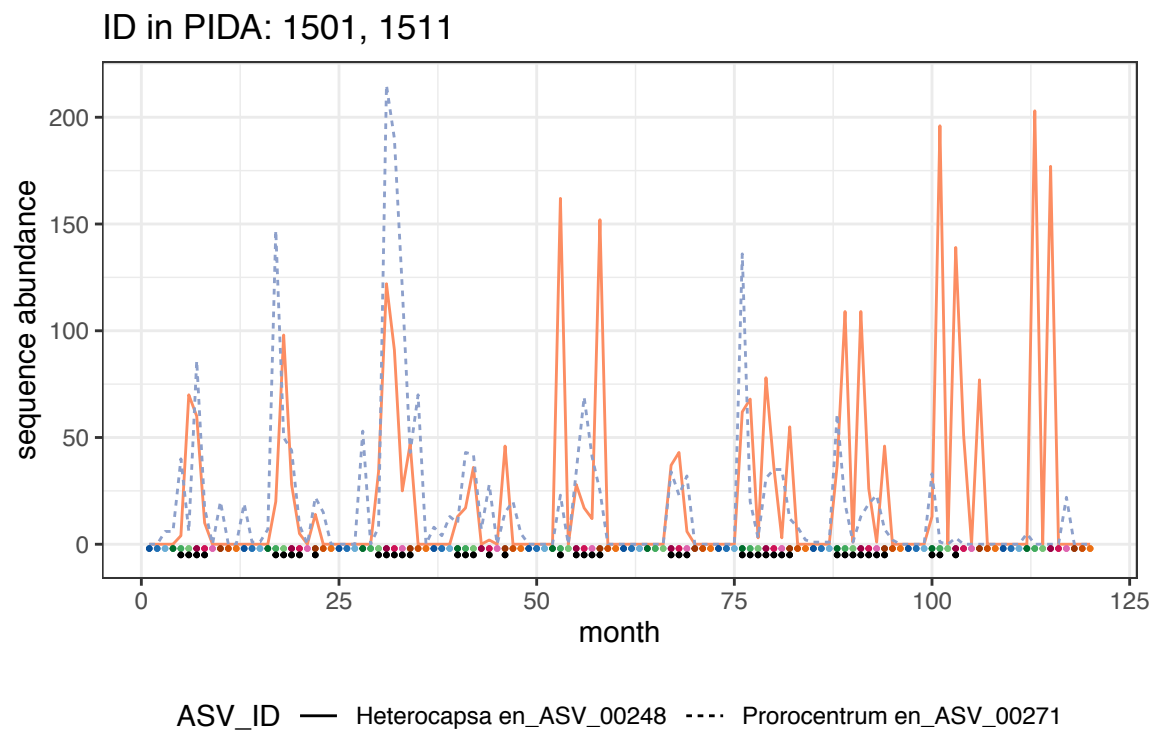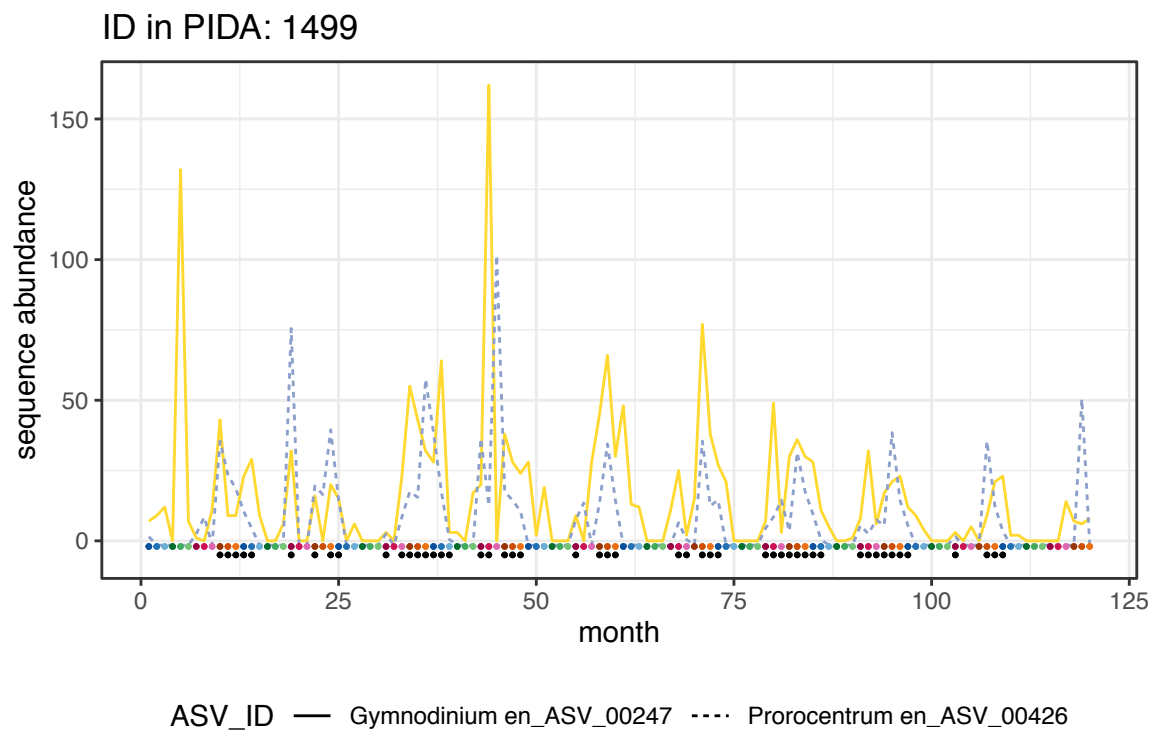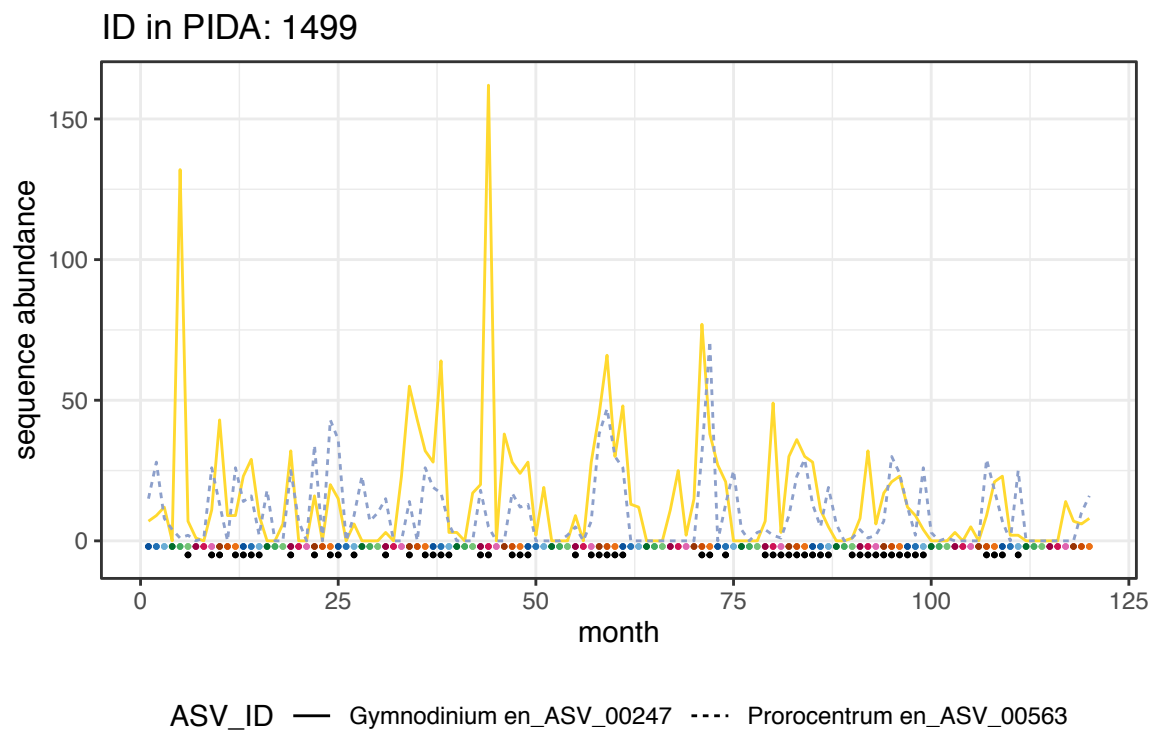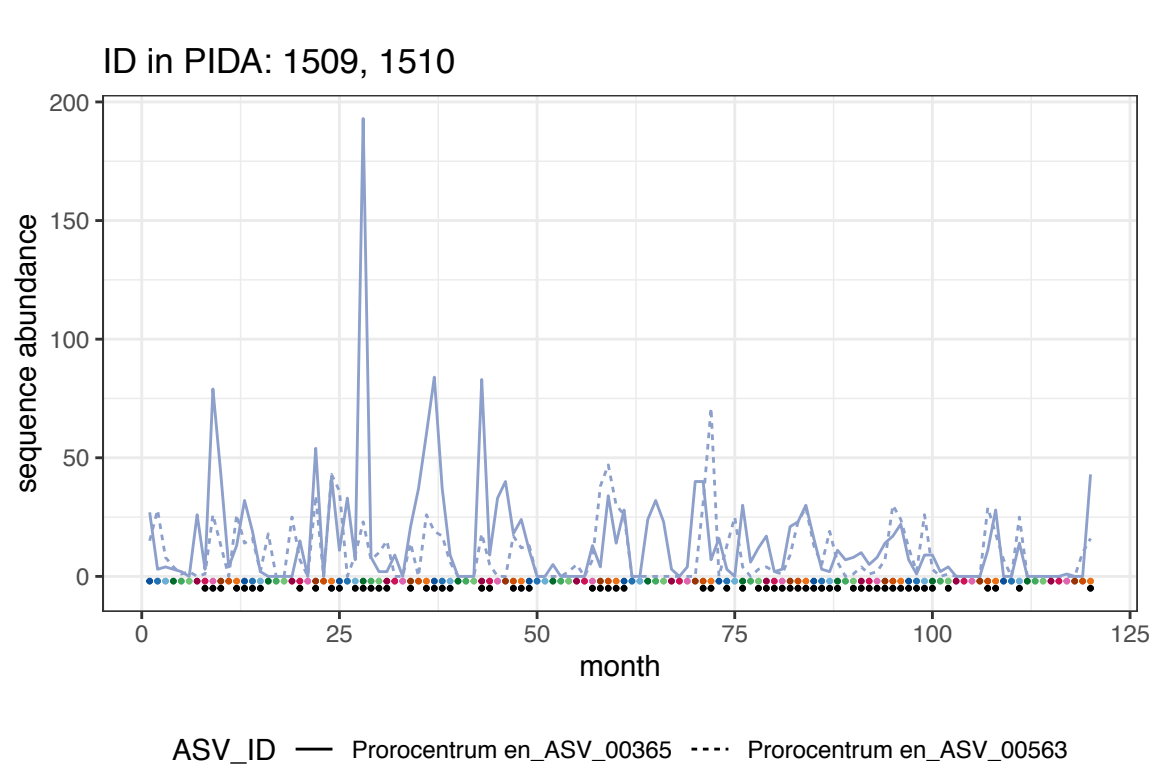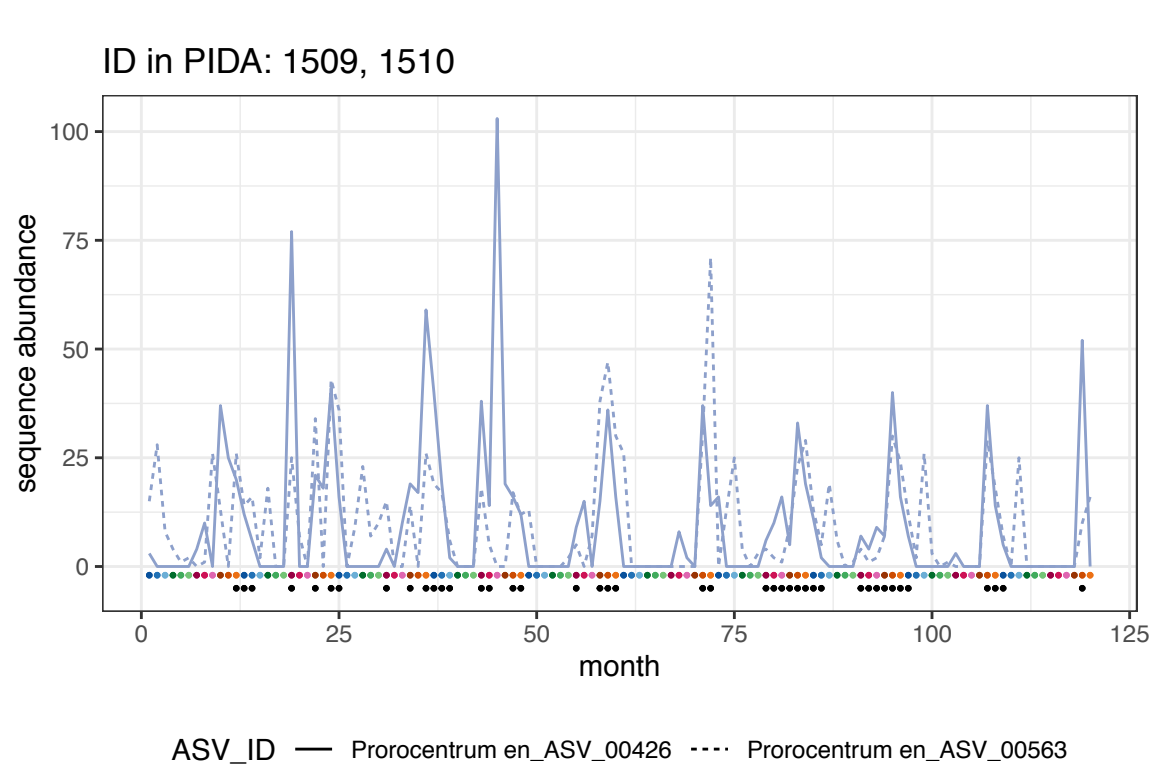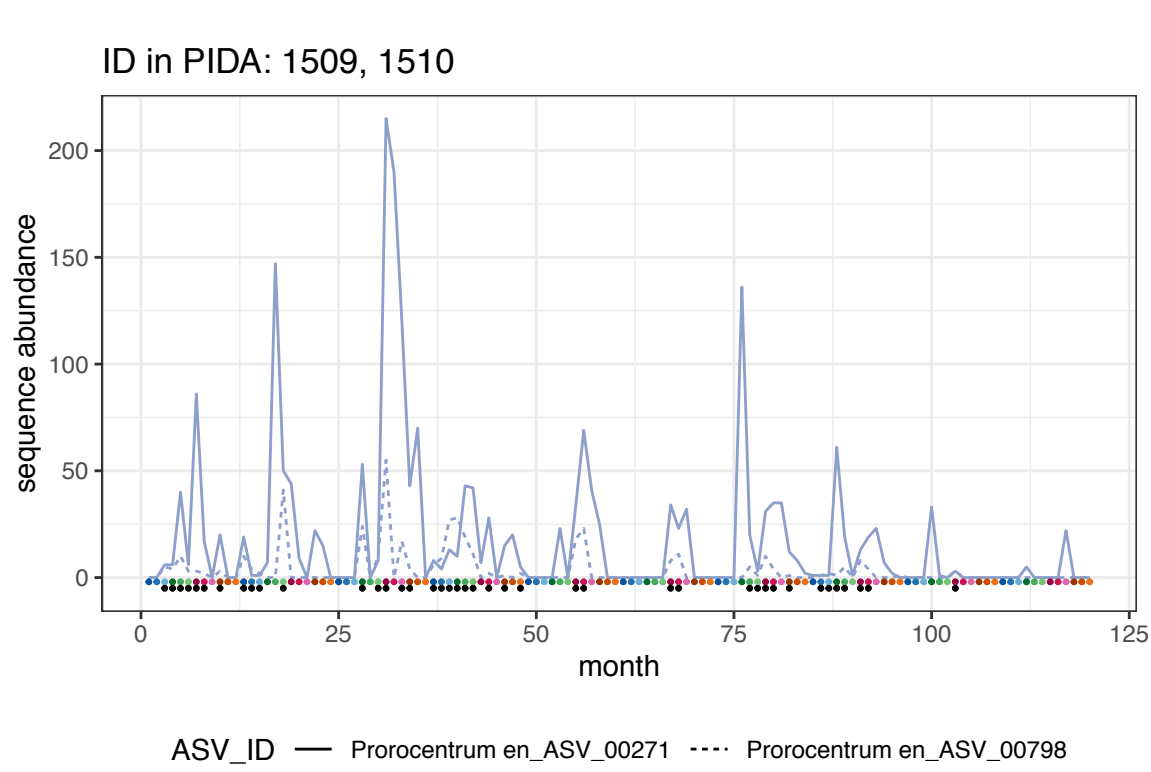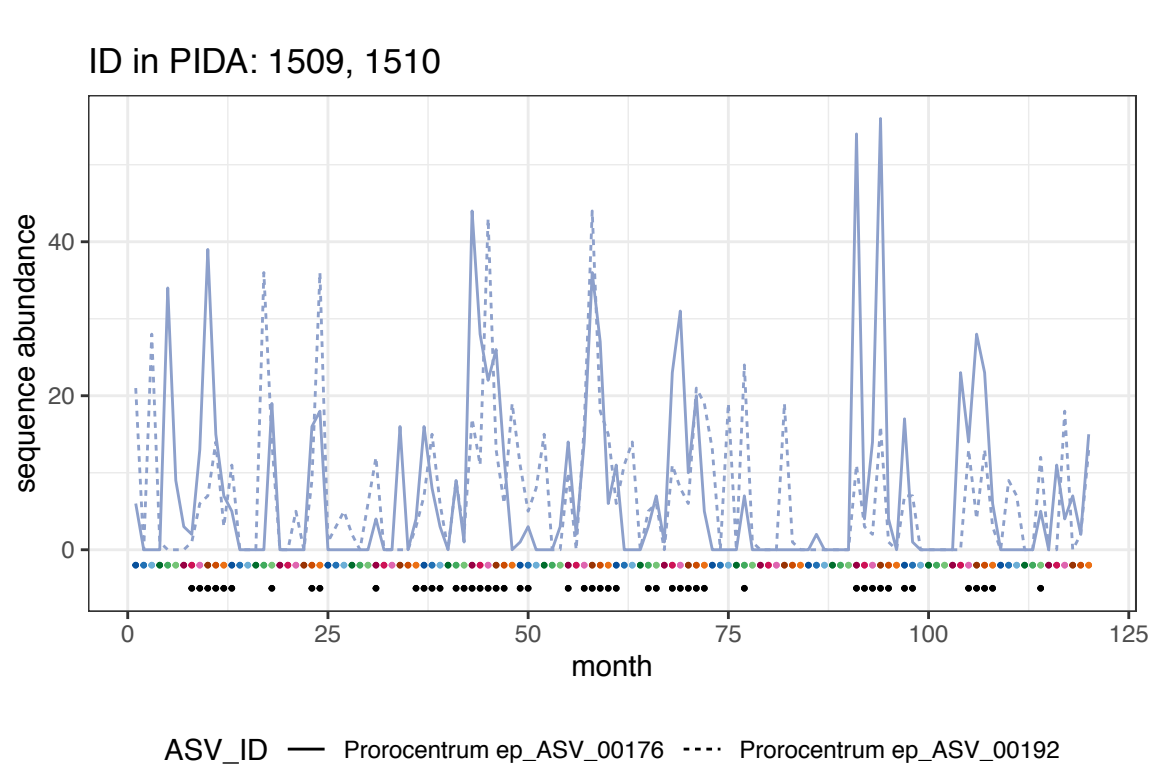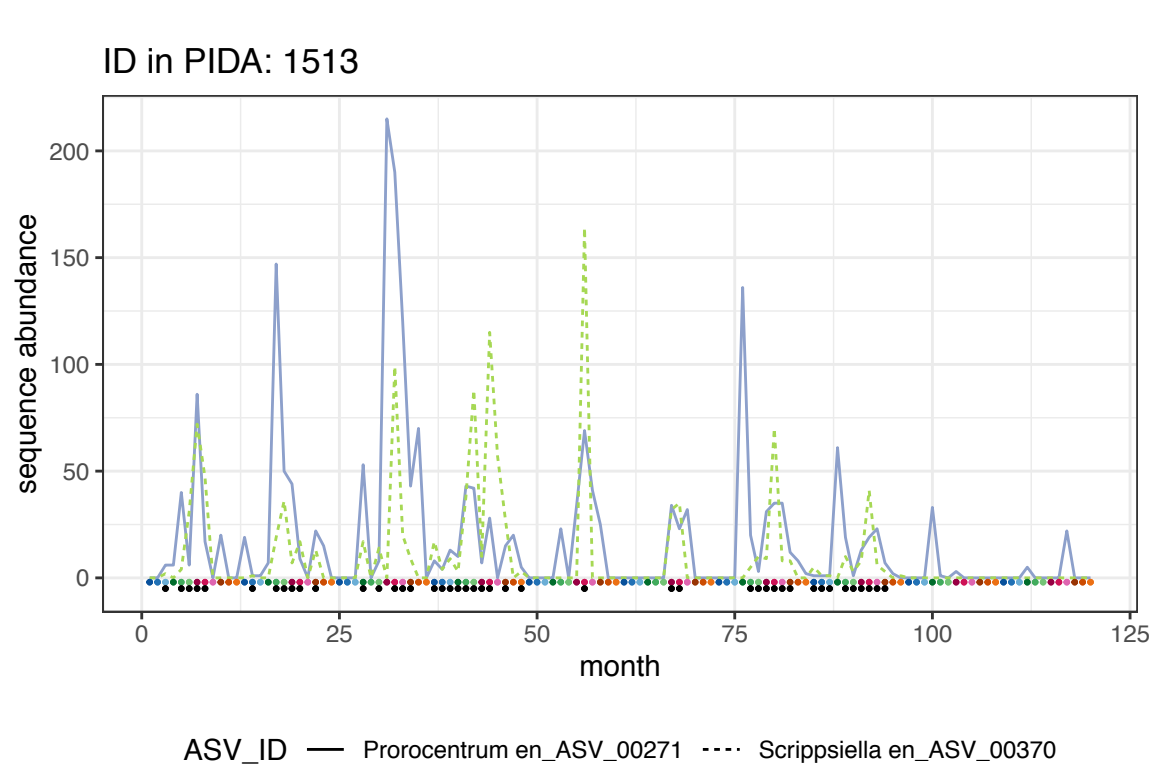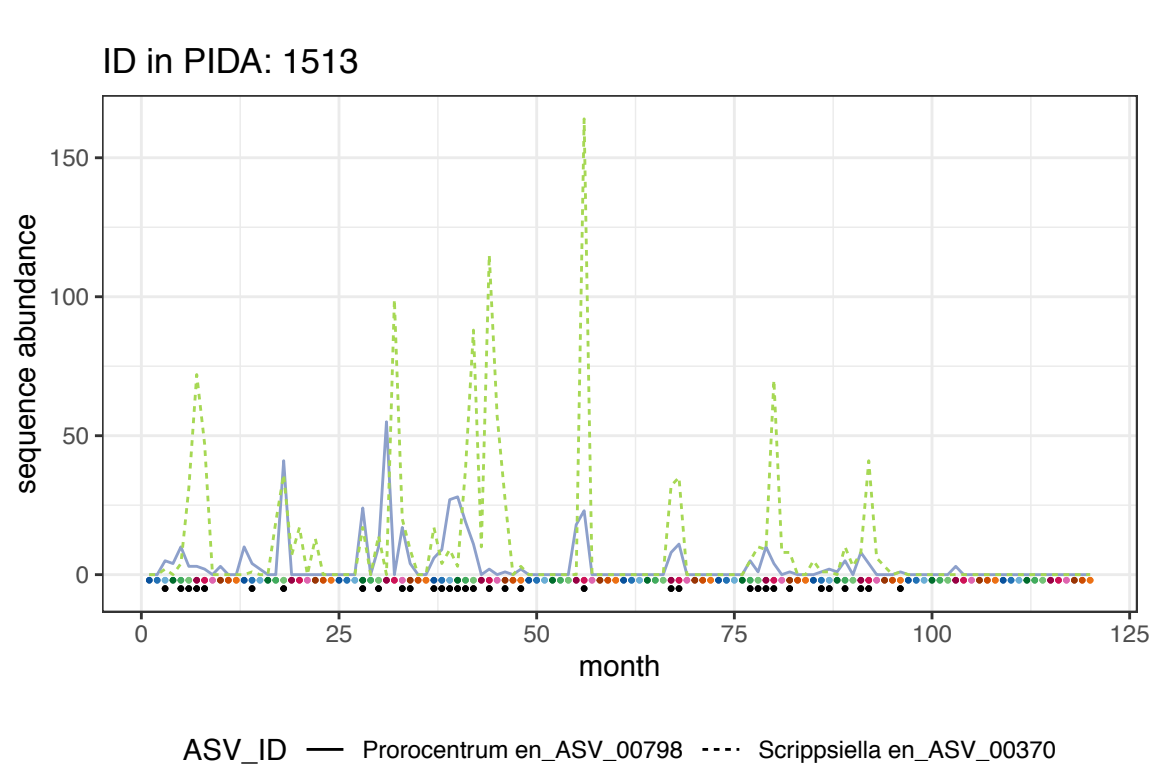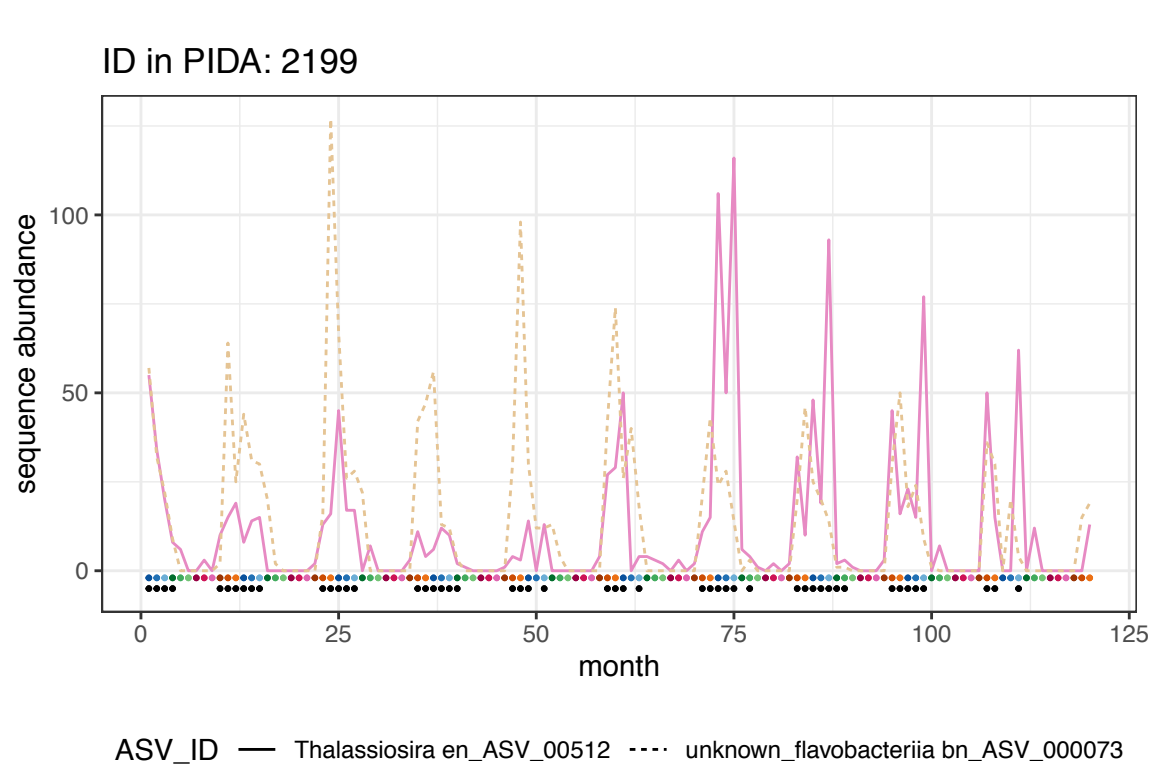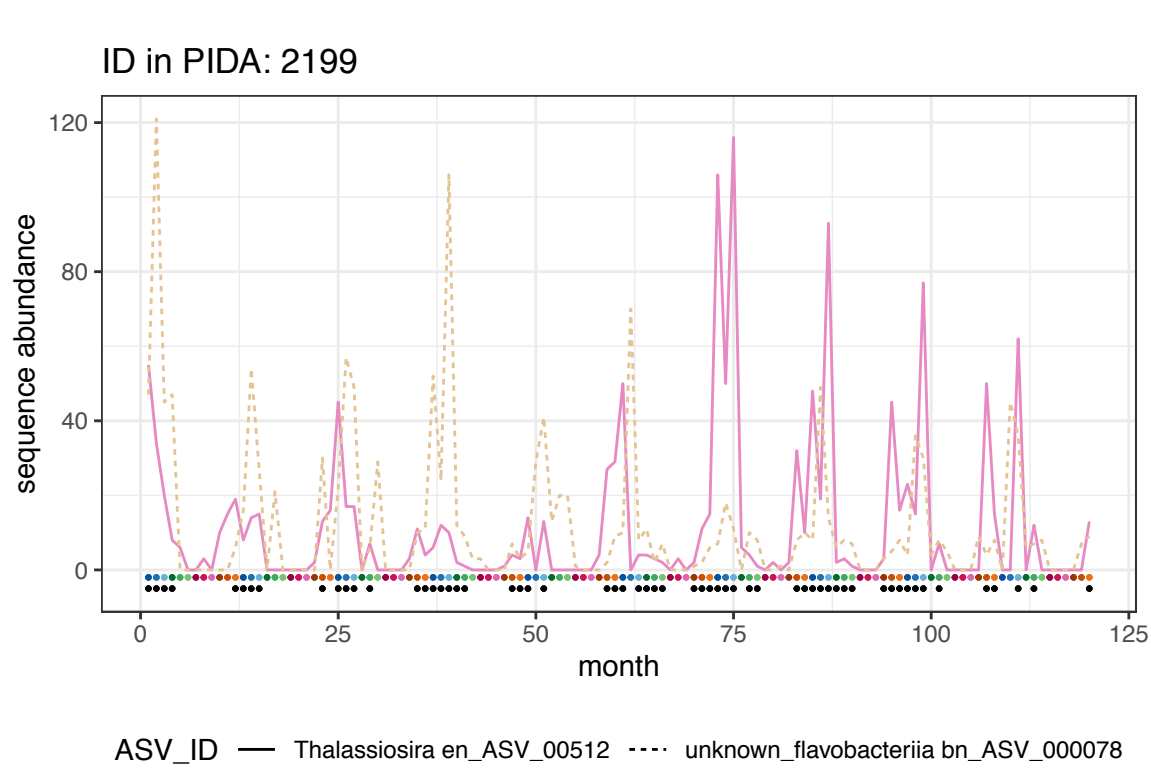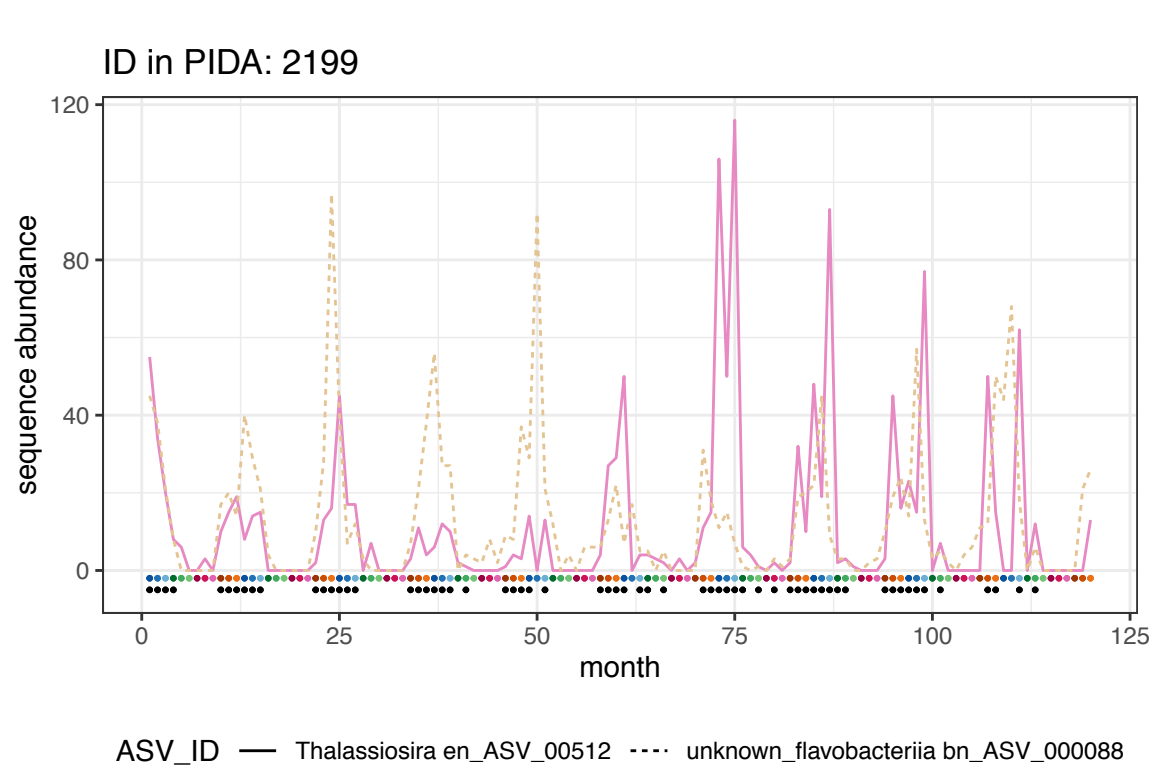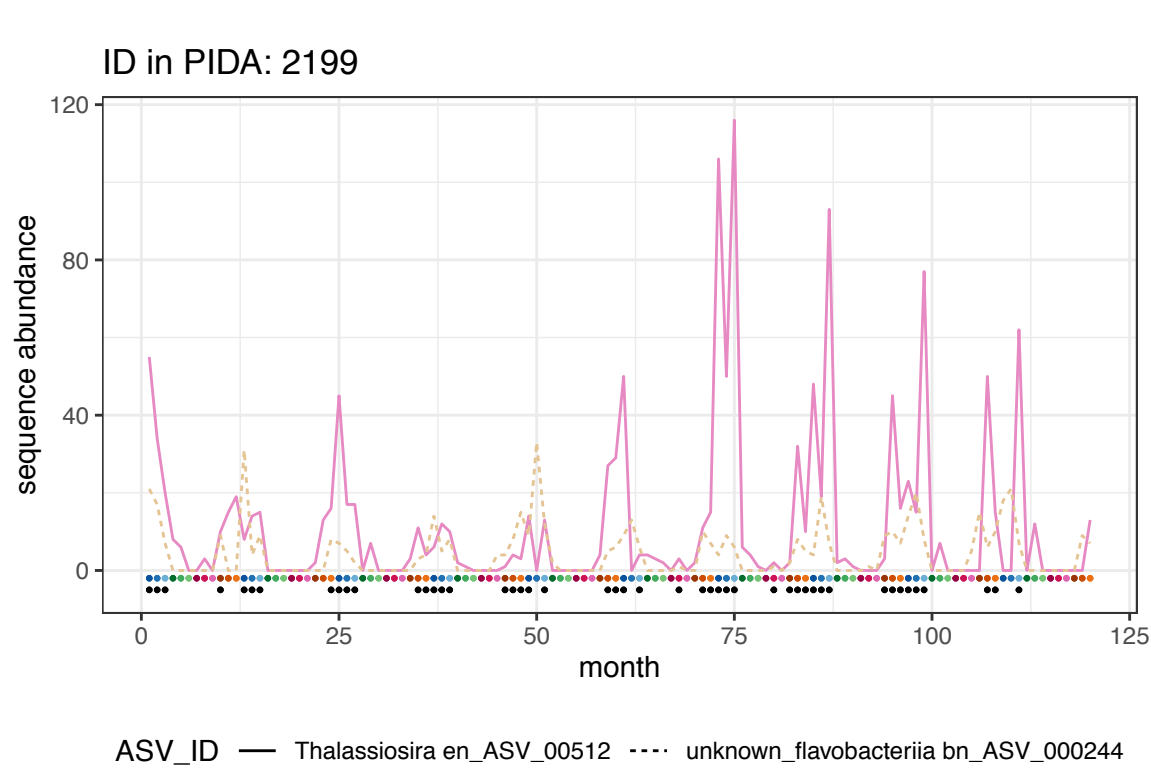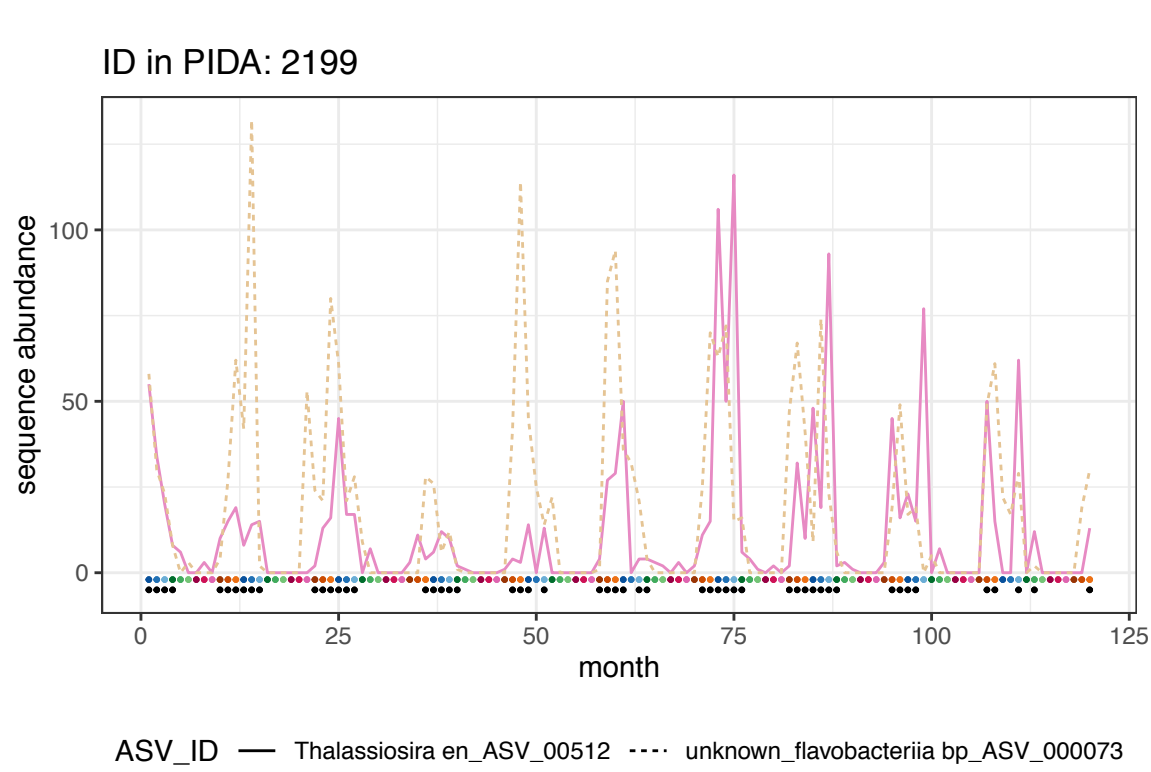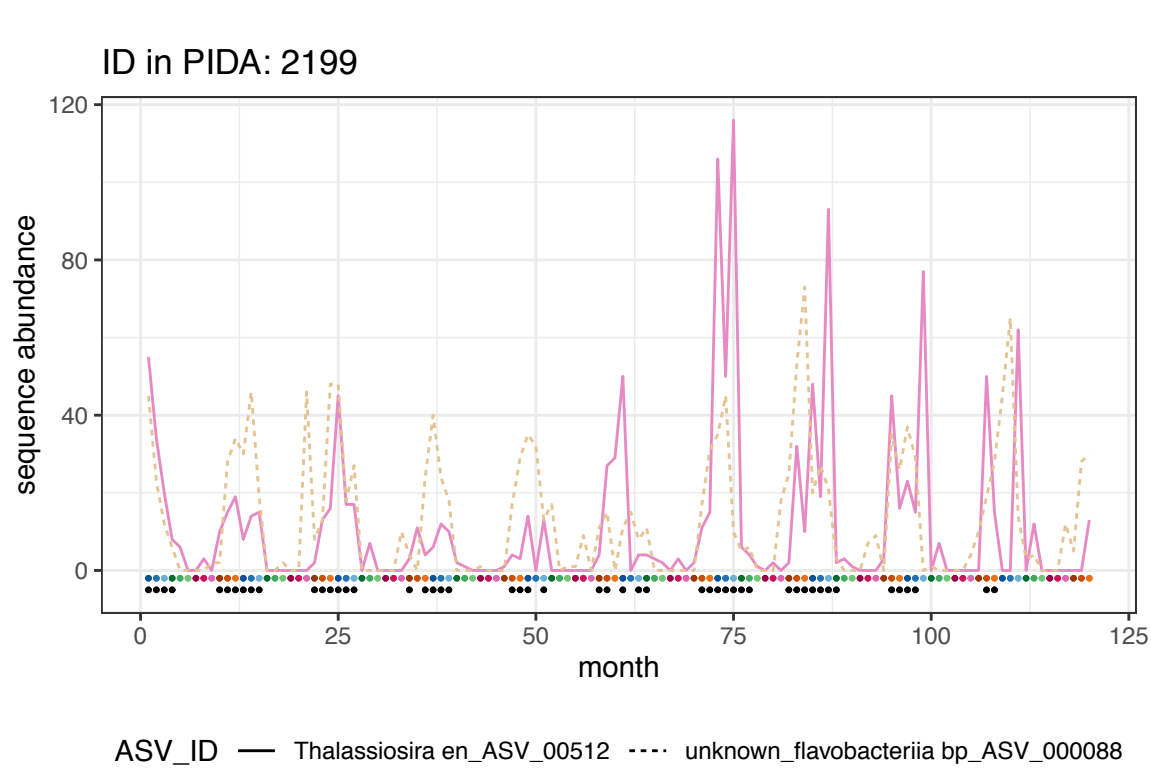

Supplement: Supplementary file 9 — Additional file 8: Supplementary Figure 8. Microbial association partners that have been reported in the literature. Found associations in the temporal network (one association per panel and a black dot on the bottom shows presence in the monthly subnetwork) and the sequence abundance in each month (solid and dashed lines). The color and line type indicate the taxonomy of the association partners. [file 40168_2023_1523_MOESM8_ESM.pdf]
