# Supplementary material for: Disentangling temporal associations in marine microbial networks
Source: Microbiome. 2023 Apr 21;11:83. doi: 10.1186/s40168-023-01523-z (PMC10120119; doi:10.1186/s40168-023-01523-z)
Supplement: Supplementary file 7 — Additional file 6: Supplementary Figure 6. Association prevalence per month. Big bar plots: distribution of associations' prevalence for each month. For example, the bar at 100 for January indicates the number of edges that have been present in all Januarys of the ten-year time series. Small bar plots: number of nodes forming the associations with a 100% prevalence. For example, only bacteria were responsible for the edges during May, with an association prevalence of 100%. Bacteria are indicated with B or b, eukaryote with E or e. ASVs from the nano size-fraction have a capital letter (B, E), and ASVs from the pico size-fraction have a small letter (b, e). [file 40168_2023_1523_MOESM6_ESM.pdf]

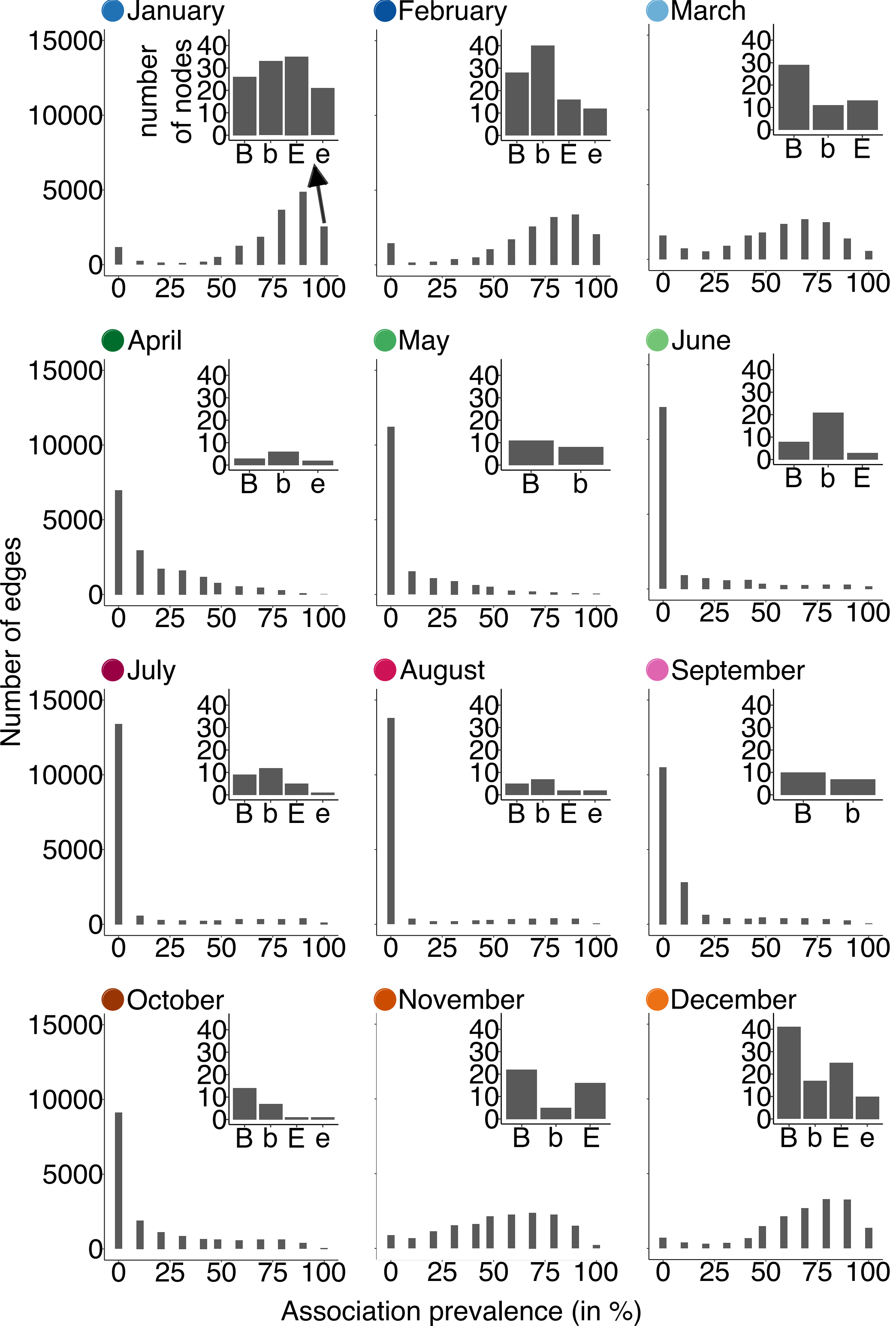

Big barplots:
associations' prevalence
within a month.

Little barplots:
number of nodes of
associations with a
100% prevalence.

B, b - Bacteria
E, e - Eukaryotes

B, E - Nano size fraction
b,e - Pico size fraction
