# Supplementary material for: Disentangling temporal associations in marine microbial networks
Source: Microbiome. 2023 Apr 21;11:83. doi: 10.1186/s40168-023-01523-z (PMC10120119; doi:10.1186/s40168-023-01523-z)
Supplement: Supplementary file 10 — Additional file 9: Supplementary Table 1. Number of nodes, removed isolated nodes, and number and fraction of edges in the preliminary network (A), and network obtained after removing environmentally-driven edges (B) and edges with association partners appearing more often alone than with the partner (C), which is the single static network. For comparison, we also give the minimum and maximum number of nodes and edges for the temporal network (D). We did not determine the union and intersection for the temporal network. If an ASV appeared in the nano and pico size fraction, it is counted twice. Therefore, for A-C) we also determined the number of microorganisms not considering size fraction (union) and being present in both size fractions (both, i.e., intersection). [file 40168_2023_1523_MOESM9_ESM.docx]

**Supplementary Table 1**: Number of nodes, removed isolated nodes, and number and fraction of edges in the preliminary network (A), and network obtained after removing environmentally-driven edges (B) and edges with association partners appearing more often alone than with the partner (C), which is the single static network. For comparison, we also give the minimum and maximum number of nodes and edges for the temporal network (D). We did not determine the union and intersection for the temporal network. If an ASV appeared in the nano and pico size fraction, it is counted twice. Therefore, for A-C) we also determined the number of microorganisms not considering size fraction (union) and being present in both size fractions (both, i.e. intersection).

|  | **A) eLSA** | **B) EnDED** | **C) Static network** | **D) Range in Temporal network** |
| --- | --- | --- | --- | --- |
| Connected nodes | 754 | 754 | 709 | 130-542 |
| Bacteria (pico) | 169 | 169 | 164 | 13-148 |
| Bacteria (nano) | 279 | 279 | 251 | 31-204 |
| Bacteria (union) | 309 | 309 | 281 |  |
| Bacteria (both) | 139 | 139 | 134 |  |
| Eukaryote (pico) | 150 | 150 | 141 | 7-124 |
| Eukaryote (nano) | 156 | 156 | 153 | 2-138 |
| Eukaryote (union) | 306 | 306 | 294 |  |
| Eukaryote (both) | 0 | 0 | 0 |  |
| Isolated nodes | 1000 | 0 | 45 | 6-38 |
| Edges | 29820 | 26505 | 16626 | 538-15083 |
| Positive edges | 24458 | 23405 | 16481 | 523-14940 |
| (%) | 82.0 | 88.3 | 99.1 | 92.2-99.7 |
| Negative edges | 5362 | 3100 | 145 | 12-143 |
| (%) | 18.0 | 11.7 | 0.9 | 0.3-7.8 |

*pico and nano – microorganism detected in the picoplankton and nanoplankton, respectively, union – how many microorganisms when not considering size-fraction, both – how many microorganims appear in both size fractions*
