# Supplementary material for: Disentangling temporal associations in marine microbial networks
Source: Microbiome. 2023 Apr 21;11:83. doi: 10.1186/s40168-023-01523-z (PMC10120119; doi:10.1186/s40168-023-01523-z)
Supplement: Supplementary file 11 — Additional file 10: Supplementary Table 2. Top 100 most prevalent/recurring associations. [file 40168_2023_1523_MOESM10_ESM.docx]

**Supplementary Table 2**: Top 100 most prevalent/recurring associations

| **Association partners** | **Number of associations** |
| --- | --- |
| Bacterial association in picoplankton | 42 |
| Bacterial association in nanoplankton | 35 |
| Bacterial associations between size fractions | 10 |
| Bacteria associated to Eukaryote in nanoplankton | 4 |
| Eukaryotic association in nanoplankton | 3 |
| Bacteria associated to Eukaryote in picoplankton | 3 |
| Bacteria in nanoplankton associated to Eukaryotic picoplankton | 2 |
| Eukaryotic association in picoplankton | 1 |
