# Supplementary material for: Disentangling temporal associations in marine microbial networks
Source: Microbiome. 2023 Apr 21;11:83. doi: 10.1186/s40168-023-01523-z (PMC10120119; doi:10.1186/s40168-023-01523-z)
Supplement: Supplementary file 12 — Additional file 11: Supplementary Table 3. Number of environmental factors leading to the removal of edges. [file 40168_2023_1523_MOESM11_ESM.docx]

**Supplementary Table 3**: Number of environmental factors leading to the removal of edges.

| **Number of environmental factors** | **Edges** | **Positive edges** | **Negative edges** |
| --- | --- | --- | --- |
| 0, i.e. not environmentally-driven edges | 26505 | 23405 (88.3%) | 3100 (11.7%) |
| 1 | 2747 | 1019 (37.1%) | 1728 (62.9%) |
| 2 | 506 | 33 (6.5%) | 473 (93.5%) |
| 3 | 61 | 1 (1.6%) | 60 (98.4%) |
| 4 | 1 | 0 (0%) | 1 (100%) |
