# Supplementary material for: Disentangling temporal associations in marine microbial networks
Source: Microbiome. 2023 Apr 21;11:83. doi: 10.1186/s40168-023-01523-z (PMC10120119; doi:10.1186/s40168-023-01523-z)
Supplement: Supplementary file 13 — Additional file 12: Supplementary Table 4. Number of environmentally-driven edges for each environmental factor and fraction considering the total number of edges (29820) in the network. In addition, we present the number of positive and negative edges and the fraction considering the number of edges removed through an environmental factor. [file 40168_2023_1523_MOESM12_ESM.docx]

**Supplementary Table 4**: Number of environmentally-driven edges for each environmental factor and fraction considering the total number of edges (29820) in the network. In addition, we present the number of positive and negative edges and the fraction considering the number of edges removed through an environmental factor.

| **Environmental factor** | **Edges** | **Positive edges** | **Negative edges** |
| --- | --- | --- | --- |
| Temperature | 1920 (6.44%) | 725 (37.8%) | 1195 (62.2%) |
| Total chlorophyll-a concentration | 838 (2.81%) | 82 (9.8%) | 756 (90.2%) |
| Day length | 730 (2.45%) | 237 (32.5%) | 493 (67.5%) |
| NO_2_^−^ | 192 (0.64%) | 26 (13.5%) | 166 (86.5%) |
| SiO_2_ | 162 (0.54%) | 6 (3.7%) | 156 (96.3%) |
| NO_3_^−^ | 57 (0.19%) | 12 (21.1%) | 45 (78.9%) |
| Turbidity | 47 (0.16%) | 0 | 47 (100%) |
| Salinity, NH_4_^+^, and PO_4_^3−^ | 0 | 0 | 0 |
