# Supplementary material for: Disentangling temporal associations in marine microbial networks
Source: Microbiome. 2023 Apr 21;11:83. doi: 10.1186/s40168-023-01523-z (PMC10120119; doi:10.1186/s40168-023-01523-z)
Supplement: Supplementary file 14 — Additional file 13: Supplementary Table 5. 100% Matching sequences from Cyanorak database for selected cyanobacterial ASVs. [file 40168_2023_1523_MOESM13_ESM.docx]

**Supplementary Table 5**: 100% Matching sequences from Cyanorak database for selected cyanobacterial ASVs

| **ASV** | **Number** | **Matching sequence name with clade and subclade** |
| --- | --- | --- |
| Synechococcus #1 | 38 | 2x A15-24 III IIIa, 2x A15-28 III IIIb, 3x A15-44 II IIa, 2x A15-62 II IIc, 2x A18-40 III IIIa, 2x A18-46.1 III IIIa, 2x BOUM118 III IIIa, 2x CC9605 II IIc, 2x M16.1 II IIa, 2x PROS-U-1 II IIh, 2x ROS8604 I Ib, 3x RS9902 II IIa, 3x RS9907 II IIa, 2x RS9915 III IIIa, 2x TAK9802 II IIa, 1x WH8016 I Ib, 2x WH8103 III IIIa, 2x WH8109 II IIa |
| Synechococcus #5 | 2 | 2x PROS-9-1 I Ib |
| Prochlorococcus #18 | 2 | 1x EQPAC1 HLI HLI, 1x MED4 HLI HLI |
| Cyanobium #20 | 2 | 1x MINOS11 5.3 5.3, 1x RCC307 5.3 5.3 |
