# Supplementary material for: Disentangling temporal associations in marine microbial networks
Source: Microbiome. 2023 Apr 21;11:83. doi: 10.1186/s40168-023-01523-z (PMC10120119; doi:10.1186/s40168-023-01523-z)
Supplement: Supplementary file 15 — Additional file 14: Supplementary Table 6. Interactions found in the BBMO temporal network that have been reported in the literature. The table shows the number of associations found in the network. For example, the association between the ASVs classified as Dia. Thalassiosira and ASVs classified as F. unknown Flavobacteriia has been found 6 times in the network. [file 40168_2023_1523_MOESM14_ESM.docx]

**Supplementary Table 6**: Interactions found in the BBMO temporal network that have been reported in the literature. The table shows the number of associations found in the network. For example, the association between the ASVs classified as *Dia. Thalassiosira* and ASVs classified as F. unknown *Flavobacteriia* has been found 6 times in the network.

| **Microorganisms** | **Occurrences** | **ID in PIDA** |
| --- | --- | --- |
| Dia. *Thalassiosira* - F. unknown Flavobacteriia | 6 | 2199 |
| Dino. *Heterocapsa* - Dino. *Prorocentrum* | 1 | 1501, 1511 |
| Dino. *Gyrodinium* - Dino. *Heterocapsa* | 1 | 1313, 1314, 1780, 1783 |
| Dino. *Prorocentrum* - Dino. *Gymnodinium* | 2 | 1499 |
| Dino. *Prorocentrum* - Dino. *Prorocentrum* | 4 | 1509, 1510 |
| Dino. *Prorocentrum* - Dino. *Scrippsiella* | 2 | 1513 |
| Abbreviations indicate Dia - Diatomea; Dino - Dinoflagellata; F - Flavobacteriia; ID in PIDA refers to the number PIDA gave to an interaction described in the literature. | | |
